# AtSNP_TATAdb: Candidate Molecular Markers of Plant Advantages Related to Single Nucleotide Polymorphisms within Proximal Promoters of *Arabidopsis thaliana* L.

**DOI:** 10.3390/ijms25010607

**Published:** 2024-01-03

**Authors:** Anton Bogomolov, Karina Zolotareva, Sergey Filonov, Irina Chadaeva, Dmitry Rasskazov, Ekaterina Sharypova, Nikolay Podkolodnyy, Petr Ponomarenko, Ludmila Savinkova, Natalya Tverdokhleb, Bato Khandaev, Ekaterina Kondratyuk, Olga Podkolodnaya, Elena Zemlyanskaya, Nikolay A. Kolchanov, Mikhail Ponomarenko

**Affiliations:** 1 Institute of Cytology and Genetics, Novosibirsk 630090, Russia; mantis_anton@bionet.nsc.ru (A.B.); ka125699ri@yandex.ru (K.Z.); filonovsv@yandex.ru (S.F.); ichadaeva@bionet.nsc.ru (I.C.); rassk@bionet.nsc.ru (D.R.); sharypova@bionet.nsc.ru (E.S.); pnl@bionet.nsc.ru (N.P.); pon.petr@gmail.com (P.P.); lksav@bionet.nsc.ru (L.S.); nata@bionet.nsc.ru (N.T.); b.khandaev@g.nsu.ru (B.K.); kandy@ngs.ru (E.K.); opodkol@bionet.nsc.ru (O.P.); ezemlyanskaya@bionet.nsc.ru (E.Z.); kol@bionet.nsc.ru (N.A.K.); 2 Natural Science Department, Novosibirsk State University, Novosibirsk 630090, Russia; 3 Institute of Computational Mathematics and Mathematical Geophysics, Novosibirsk 630090, Russia; 4 Siberian Federal Scientific Centre of Agro-BioTechnologies of the Russian Academy of Sciences, Krasnoobsk 630501, Novosibirsk Region, Russia

**Keywords:** TATA-binding protein, TATA box, noncoding polymorphism, gene expression, estimates in silico, genome-wide analysis, verification in vivo, target-assisted breeding

## Abstract

The mainstream of the post-genome target-assisted breeding in crop plant species includes biofortification such as high-throughput phenotyping along with genome-based selection. Therefore, in this work, we used the Web-service Plant_SNP_TATA_Z-tester, which we have previously developed, to run a uniform in silico analysis of the transcriptional alterations of 54,013 protein-coding transcripts from 32,833 *Arabidopsis thaliana* L. genes caused by 871,707 SNPs located in the proximal promoter region. The analysis identified 54,993 SNPs as significantly decreasing or increasing gene expression through changes in TATA-binding protein affinity to the promoters. The existence of these SNPs in highly conserved proximal promoters may be explained as intraspecific diversity kept by the stabilizing natural selection. To support this, we hand-annotated papers on some of the Arabidopsis genes possessing these SNPs or on their orthologs in other plant species and demonstrated the effects of changes in these gene expressions on plant vital traits. We integrated in silico estimates of the TBP-promoter affinity in the AtSNP_TATAdb knowledge base and showed their significant correlations with independent in vivo experimental data. These correlations appeared to be robust to variations in statistical criteria, genomic environment of TATA box regions, plants species and growing conditions.

## 1. Introduction

Biofortification combines high-throughput phenotyping along with genome-based acceleration of selection [1]. This becomes a mainstream of the targeted breeding in agricultural plants [1]. Among whole-genome technologies, CRISPR/Cas-mediated editing of cis-regulatory elements is the most widespread for crop improvement [2,3,4]. For genome-editing of TATA-binding protein (TBP)-binding sites (TBP-sites) [5], the most evolutionally conservative [6] and obligatory [7,8] transcription initiation site in eukaryotic promoters [9,10], a *Faecalibaculum rodentium* Cas9 protein was recently discovered [5]. Moreover, all things being equal, the expression level of a gene increases with an increase in the TBP binding affinity for its 90 bp proximal promoter [11,12]. Additionally, the consensus for plant TBP-sites—i.e., tcacTATATATAg within (A+T)-rich local contextual environment—has been commonly accepted for more than a quarter of a century [13], in contrast to the TATA(a/t)A(a/t)g consensus of this site in the (G+C)-rich environment in animals [14]. The 500 bp regions just upstream of the transcription start sites (TSSs) are the commonly accepted promoters of the *Arabidopsis thaliana* genes because of their enrichment in *cis*-regulatory elements and depletion in single nucleotide polymorphisms (SNPs) [15]. In natural ecotypes of *Arabidopsis*, SNPs in the promoter regions between positions −40 and +40 relative to TSSs of the protein-coding genes affect expression of these genes [16]. In addition, SNPs in *Arabidopsis* promoters can change transcription start sites, binding of transcription factors, as well as the epigenetic status of promoters [17,18,19,20,21]. For example, the majority of natural accessions (>88%) have G at position −230 relative to the TSS of a floral repressor gene *FLOWERING LOCUS C (FLC)* and autumnal (high) expression in response to winter cold. On the other hand, A at this position promotes the usage of the alternative upstream (−100) transcription start site (uTSS) instead of the major one (mTSS) and by this reduces FLC expression and accelerates flowering [22]. The SNPs are unevenly distributed near the TSS; for example, in rice, the decline in SNP density was recorded at −250 bp, and this decline comes to a minimum at the TSS [23].

The 90 bp proximal promoter region covers all experimentally proven natural TBP-sites in eukaryotes, and we have previously created a Web service Plant_SNP_TATA_Z-tester (http://wwwmgs.bionet.nsc.ru/cgi-bin/mgs/tatascan_plant/start.pl, accessed 20 June 2023) for calculating in silico the effects of SNPs within the 90 bp proximal promoter region at the gene expression level [24]. It implements a three-step model of TBP-promoter molecular binding [25] such as (i) TBP slides along the promoter DNA-helix [26] ↔ (ii) TBP stops at a potential TBP-binding site (TBP-site) [6,27] ↔ (iii) the TBP–promoter complex is stabilized due to DNA bending at a 90° angle [28], as observed experimentally in vitro [29]. Additionally, the results of the Plant_SNP_TATA_Z-tester were successfully verified using twelve independent experiments measuring the effects of single, paired and multiple nucleotide substitutions, deletions and insertions, as well as artificial promoter constructs on the expression of tobacco and *Arabidopsis* genes in vivo, ex vivo and in vitro [24]. Finally, as a practical Plant_SNP_TATA_Z-tester implementation, we have discovered the fact of spontaneous selection in ancient times by farmers of the crop plants with reduced expression of genes encoding food allergens, namely β-amylase, albumin and globulin [30].

In the present work, using the Plant_SNP_TATA_Z-tester [24] we conducted the genome-wide in silico analysis of 871,707 *Arabidopsis* SNPs within 90 bp proximal promoters versus 48,321 protein-coding transcripts from 32,833 genes and calculated their effects in gene expression. We predicted 54,993 SNPs to decrease or increase TBP affinity to promoters and, consequently, gene expression. We illustrated the influence of some of the genes possessing these SNPs on plant development and responses to environmental changes with independent published experimental data in vivo and annotated the impact of some of them to plant vital traits.

## 2. Results and Discussion

### 2.1. The Genome-Wide Prediction of SNPs in Proximal Promoters

**Figure 1:**
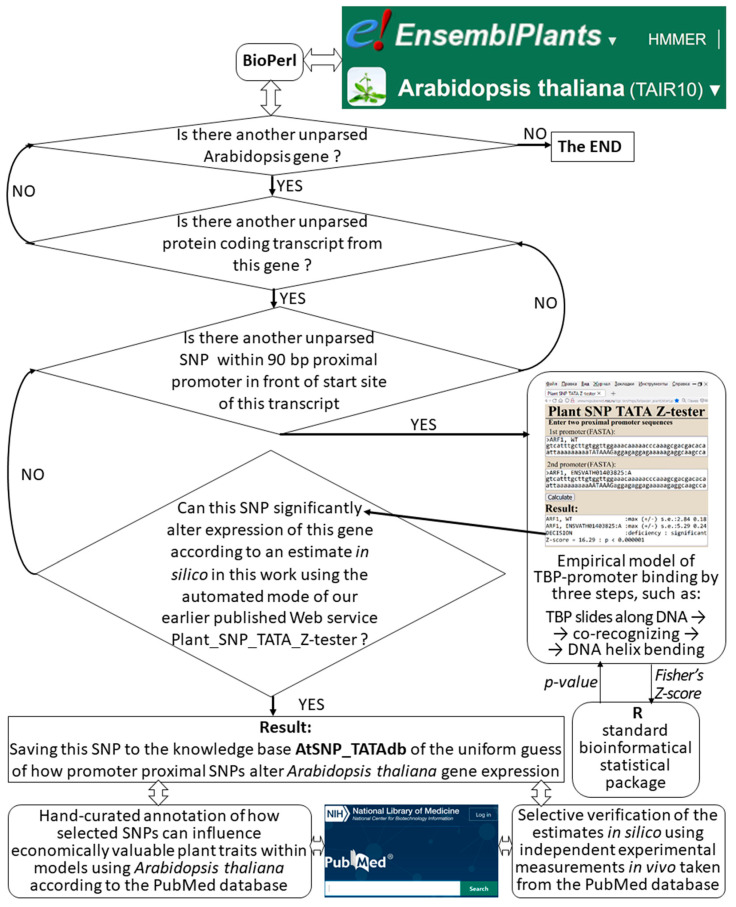
Flowchart of AtSNP_TATAdb knowledge base development by processing genome-wide information from the Ensembl Plant database [31] by first using Plant_SNP_TATA_Z-tester [24] and, after that, selectively annotating some of the SNPs by a search in the PubMed database [39] using information about where, when and under what conditions changes in expression of these genes or their homolog genes were observed in agricultural studies. Finally, we selectively verified estimates in silico using independent experimental data in vivo taken from the PubMed database [39].

In this work, we identified in silico in the *A. thaliana* genome the proximal SNPs in promoters of protein-coding genes for the first time, which can significantly alter the TBP binding affinity, as schematically shown in Figure 1 and quantitatively characterized in Table 1. First of all, among Arabidopsis genes annotated in the Ensembl Plants database [31] (accessed on 16 October 2023), we selected 27,628 protein-coding genes (80%) as well as 48,321 protein-coding transcripts among 54,013 transcripts according to the current state of this database (Table 1: 89.5% of 100%). Then, using the automatic mode of Plant_SNP_TATA_Z-tester [24] (hereinafter, see “Supplementary Materials” [25,26,27,28,29,32,33,34,35,36,37,38], Section S1), we examined separately, one-by-one each of 871,707 single nucleotide substitutions (SNPs) localized within the 90 bp proximal region adjacent to the TSS of each transcript.

As a result, 54,993 candidate SNP markers (6.3%) were identified as having significant changes in the TBP affinity for the promoters, namely 27,425 (3.2%) and 27568 (3.2%) SNPs, minor alleles of which significantly downregulate and upregulate the 18,636 *Arabidopsis* genes (56.78%), respectively.

**Figure 2:**
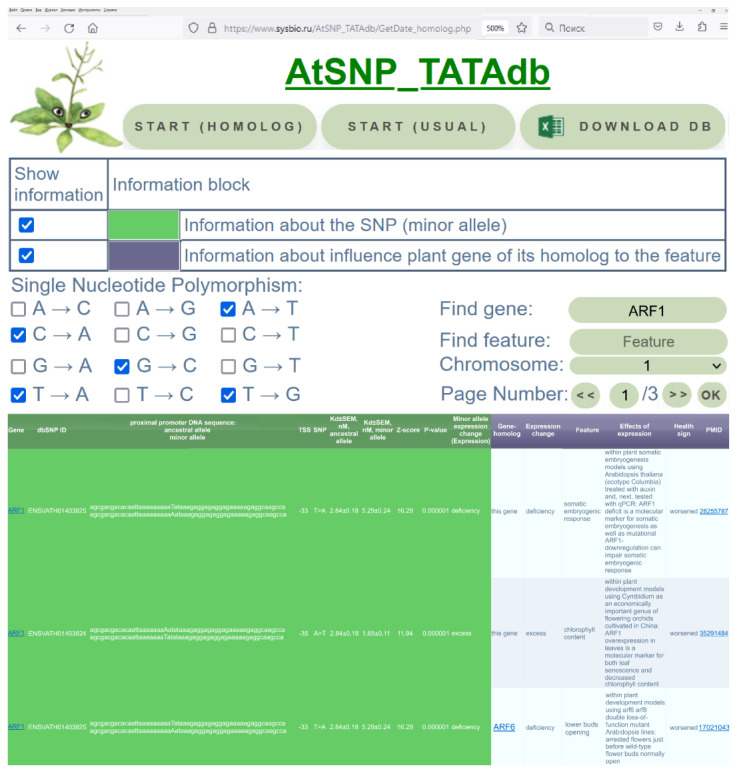
A sample entry in the AtSNP_TATAdb documents on two SNPs ENSVATH01403825:A and ENSVATH01403824:T of the *ARF1 (Auxin Response Factor 1*) gene, which can significantly downregulate and upregulate its expression, as calculated in this work together with their annotation (see Supplementary Materials, Table S1: first row) using the database PubMed [39] (PMIDs: 28255787, 35291484, 17021043).

Finally, we saved these results in the database AtSNP_TATAdb, which was developed in this work as described in the “materials and methods” section and is illustrated in Figure 2. Clicking on the middle “Start (usual)” button in the interface depicted in Figure 2, one can find all these genome-wide results freely available (https://www.sysbio.ru/AtSNP_TATAdb/).

### 2.2. Genome-Wide Statistical Analysis of How SNPs Can Affect TBP Binding to the Proximal Promoters of Arabidopsis Protein-Coding Genes

In rows 10 to 16 (Table 1), the readers can see the results of genome-wide statistical analysis of how all currently known SNPs located in the proximal promoters of protein-coding genes can affect TBP binding. First of all, within in silico calculations, the arithmetic mean and its standard error rate for the equilibrium dissociation constant (K_D_) of the complexes between the TBP and only ancestral alleles of the promoters (i.e., Mean ± SEM) equals 3.96 ± 0.01 nM. Additionally, in the case of only the minor alleles, this estimate was 4.07 ± 0.01 nM. Taking into account the Bonferroni correction for multiple comparisons, this difference between ancestral and minor SNP alleles is significant according to both the Student’s *t*-test (*t* = 8.07, P_ADJ_ < 10^−7^) and the Fisher’s Z-test (Z = 7.78, P_ADJ_ < 10^−2^).

Moreover, the standard deviation of K_D_ values from their arithmetic mean estimated for minor variants unexpectedly turned out to be significantly less than that for ancestral variants of the same promoters by means of the Fisher’s F-test (respectively: 1.90 nM < 1.95 nM and F = 1.06, P_ADJ_ < 10^−2^). Finally, the significance of the differences between the distributions of K_D_-values for ancestral and minor alleles was also confirmed by both the Pearson chi-square (*χ*^2^ = 298.17, P_ADJ_ < 10^−9^) and Kolmogorov–Smirnov tests (*D* = 0.04, P_ADJ_ < 10^−9^).

**Figure 3:**
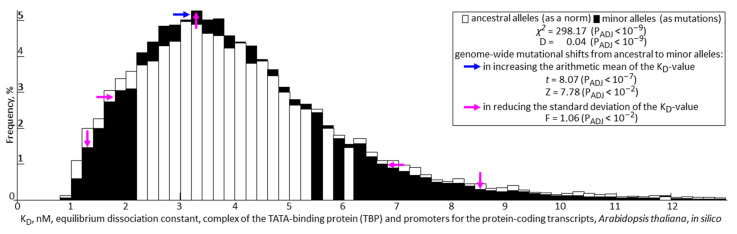
A comparison between the distributions of the K_D_-values calculated in silico using ancestral and minor alleles of the *Arabidopsis* SNPs. *Legend*: *χ*^2^ and D, the scores of Pearson’s chi-square test and Kolmogorov–Smirnov’s test for assessing the difference between two distributions, respectively; *t* and Z, the scores of Student’s *t*-test and Fisher’s Z-test for comparing the difference between two estimates of arithmetic means, respectively; F, Fisher’s F-test for comparing the difference between two estimates of the standard deviation; P_ADJ_, statistical significance level with the Bonferroni correction for multiple comparisons calculated using STATISTICA version 10.0 (Statsoft^TM^, Tulsa, OK, USA).

Figure 3 illustrates this result by distributions of the K_D_-values calculated in silico using separately either ancestral or minor alleles of the SNPs. The blue-colored right arrow (**→**) in this figure indicates a mutational shift from ancestral to minor alleles in the direction of increasing K_D_-values that corresponds to a decrease in the TBP binding affinity for minor versus ancestral alleles. The purple-colored arrows (→) symbolize mutational shifts in the direction of the reducing standard deviation of K_D_-values from ancestral to minor alleles that might be consistent with both types of alleles being under pressure of natural selection leading to stabilization.

**Figure 4:**
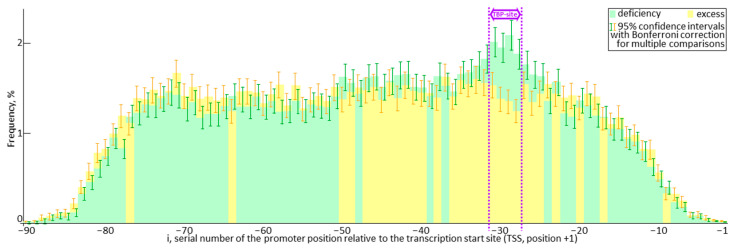
The frequencies of occurrence along promoters of candidate SNP markers that increase versus decrease the expression of protein-coding genes in Arabidopsis. *Legend*: error bars: the 95% confidence interval boundaries with the Bonferroni correction for multiple comparisons calculated using STATISTICA version 10.0 (Statsoft^TM^, Tulsa, OK, USA).

Because natural selection is usually associated with the vital traits of living organisms, it seems necessary to discuss these mutational shifts in the K_D_-values in more detail. Figure 4 specifies the predominant decrease in ratings in silico for the gene expression in the case of minor alleles of their proximal promoters in comparison with that for the ancestral ones. In this figure, light gray and dark gray bars at each position between −90 and −1 relative to the TSS (X-axis) display the frequency rates of SNP occurrence (Y-axis) for statistically significant decreases and increases in the expression of these genes.

First of all, taking into account the 95% confidence intervals of these frequencies by means of the Bonferroni correction for multiple comparisons (error bars, “I”), there is only a region in the promoters where SNP-caused damage of TBP sites significantly prevails over those improvements of these sites (Figure 4: double-headed arrow “TBP-site” in-between two purple-colored broken lines). This may correspond to preferential mutational damage of TBP binding to minor alleles compared to the ancestral alleles as a somewhat current norm. In Figure 3, this might correspond to two purple-colored down (“↓”) and right (“→”) arrows above the X-axis region in-between 0 nM and 3 nM.

Additionally, in promoters, this TBP-site region is immediately adjacent to the area of a position-by-position trend towards SNP-related down-regulation of expression that is next followed by the area with a trend towards SNP upregulation of these genes. These latter regions of mutational improvement in TBP binding to minor alleles with respect to their ancestral norm could correspond in Figure 3 to another pair of purple-colored down (“↓”) and right (“→”) arrows, which characterize the region of the X axis where K_D_-values exceed 5 nM.

Thus, the only purple upward arrow (“↑”) in this figure summarizes these two opposing SNP-dependent trends in weakening the strong TBP binding at TBP sites and strengthening the weak binding of TBP to the surrounding area of these sites that forms the mutational decrease in the standard deviation of K_D_-values identifying the stabilizing natural selection in favor of vital plant traits.

Finally, the reader can find in this figure the leftmost and rightmost regions, approximately 20 bp long, within the boundaries of the proximal promoters, where the frequencies of SNPs that can significantly change TBP-promoter binding decrease with decreasing distance to the boundaries of the proximal promoters [12].

### 2.3. Manually Curated Annotation of the Promoter Proximal SNPs

**Figure 5:**
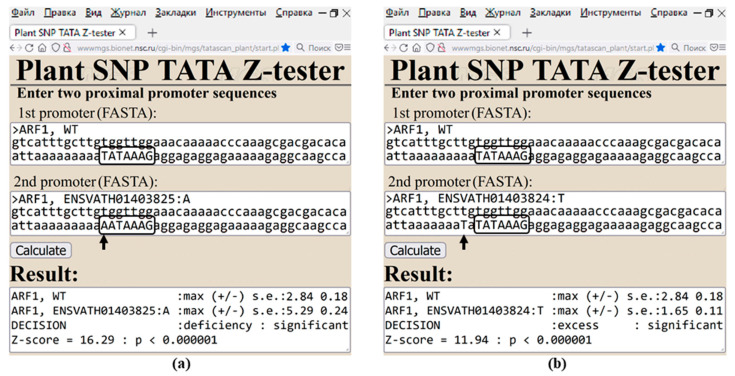
The output of Web service Plant_SNP_TATA_Z-tester [24] regarding assessment of SNPs (**a**) ENSVATH01403825:A and (**b**) ENSVATH01403824:T located in the proximal promoter of the *Arabidopsis* gene *ARF1*. One can see the results along with their annotations in Figure 2 as an illustration of how to use the knowledge base AtSNP_TATAdb.

To understand the unexpectedly discovered mosaic structure of the proximal promoters, we manually verified our calculations for the *ARF1* gene, as shown in Figure 5. This figure represents two SNPs, ENSVATH01403825:A and ENSVATH01403824:T, which likely correspond to downregulation and upregulation of this gene, as calculated in silico.

On one hand, the first SNP (i.e., ENSVATH01403825:A) is the substitution “T => A” at position −33 relative to the transcription start site of ARF1-204 annotated in the Ensembl Plant database [31], such as “taaaaaaaaaTATAAAGagga => taaaaaaaaaAATAAAGagga” (Figure 5a, Table S1 [37,38,39,40,41,42,43,44,45,46,47,48,49,50,51,52,53,54,55,56,57,58,59,60,61,62,63,64,65,66,67,68,69,70,71,72,73,74,75,76,77,78,79,80,81,82,83,84,85,86,87,88,89,90,91,92,93,94,95,96,97,98,99,100,101,102,103,104,105,106,107,108,109,110,111,112,113,114,115,116,117,118,119,120,121,122,123]). As readers can see, this substitution disrupts the canonical TATA box “TATAAAAG” (in CAPITAL font) and thus increases the K_D_-value from 2.84 ± 0.18 to 5.29 ± 0.24, reducing the affinity of TBP for this promoter, which can ultimately lead to ARF1 deficiency, the estimation of which is significant according to the Fisher’s Z-test (Z = 16.29, *p* < 0.000001).

On another hand, the second SNP (i.e., ENSVATH01403824:T) is the substitution “A => T” at position −35 relative to the same transcription start site of the ARF1 gene, such as “aattaaaaaaaAaTATAAAGag => aattaaaaaaaTaTATAAAGag” [31] (Figure 5b and Table S1). According to our calculations, this mutation located within the (A+T)- rich area in front of the same canonical TATA box decreases the K_D_-value from 2.84 ± 0.18 to 51.65 ± 0.11 that can increase both the affinity of TBP for this promoter and the *ARF1* expression (Figure 5b and Table S1: Z = 11.94, *p* < 0.000001).

This example allows us to heuristically assume that SNPs within TBP-binding sites may more often damage these sites and, therefore, reduce the expression of the corresponding genes, in contrast to SNPs in the local (A+T)-rich environment of TBP sites, which may more often improve both TBP-promoter affinity and gene expression. With this in mind, multiple repetition of this effect along with varying the positions of TBP sites from promoter to promoter can give an interference pattern of alternating areas within proximal promoters, which predominantly trend towards SNP-related upregulation and downregulation of gene expression (Figure 4). This can reduce the standard deviation of the K_D_-values of TBP complexes with minor alleles (Table 1: row #15) and cause the discussed stabilizing natural selection in favor of vital plant traits (Figure 3).

With this in mind, using the PubMed database [39], we learned that both increases and decreases in ARF1 gene expression relative to the ancestral optimum, negatively affect plant development as impaired somatic embryogenesis in *A. thaliana* [40] and reduced chlorophyll content in *Cymbidium goeringii* [41] (Figure 2 and Table S1). The existence of SNPs in very conserved proximal promoters, with their ability to equally likely increase and decrease the expression of plant genes keeping them around the evolutionarily fixed optimal expression level, may indicate stabilizing natural selection in favor of intraspecific biodiversity as the gold standard for the survival of both populations and species [124].

We also described the possible effect of increased or decreased expression for another 109 genes from having SNPs in proximal promoters by the hand-curated annotation (Table S1) of 83 original articles and found 173 putative advantages (Table 1) such as fast growing [43], lateral root formation [48], inorganic phosphate deficiency stress response [52], antiviral response [59], photo-oxidative stress response [61], food allergenicity [83], susceptibility to parasites [89], flowering time [92], trichome number [97], plant–environment response [105], seed mineral concentrations [108], drought stress response [111], photosynthetic efficiency [113], cold tolerance [116], coenzyme Q10 concentration [118] and resistance to low-nutrient conditions [123]. These vital plant traits can vary depending on the proximal promoter SNPs, as observed experimentally in 17 crop species (Table 1) such as *Cymbidium goeringii* [41], *Triticum turgidum* [51], *Camellia oleifera* [52], *Solanum lycopersicum* [57], *Vitis vinifera* L. [58], *Oryza sativa* L. [59], *Carica papaya* [60], *Betula pendula* [69] and *Lilium longiflorum* [72] (Table S1).

All these data are freely available as our knowledge base AtSNP_TATAdb (https://www.sysbio.ru/AtSNP_TATAdb/), as accessed on 15 December 2023.

### 2.4. Validation of In Silico Estimates of KD-Values Documented in the AtSNP_TATAdb Knowledge Base Using Independent In Vivo Experimental Data

**Figure 6:**
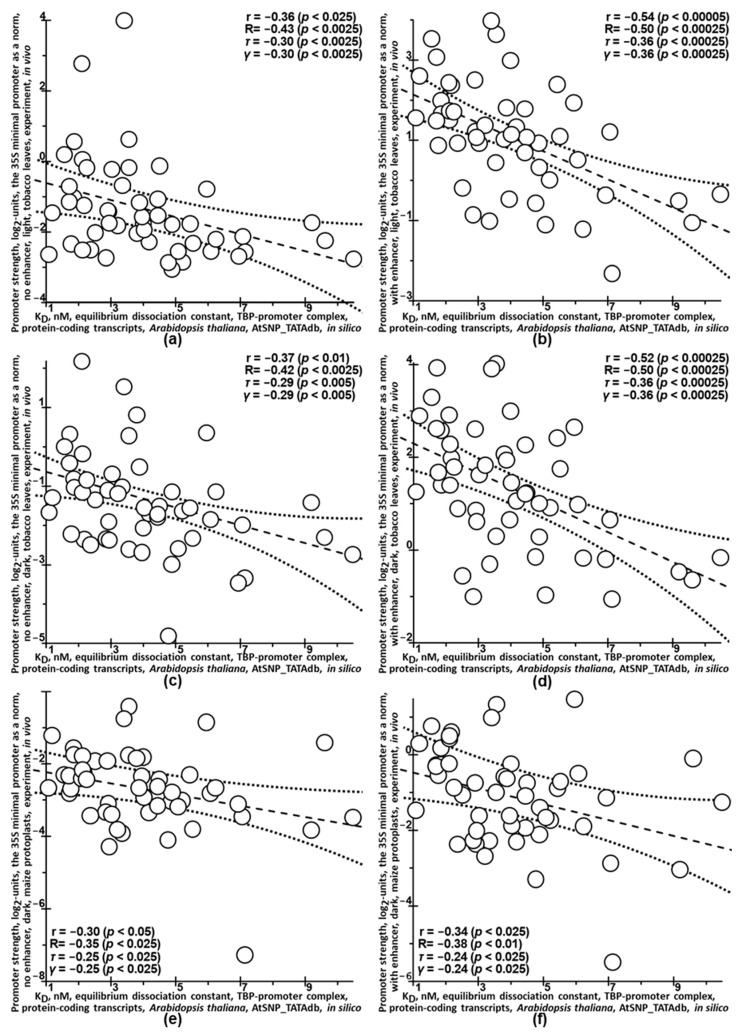
Statistically significant correlations between the K_D_-values of the equilibrium dissociation constant expressed in nanomoles per liter (nM) of the complexes between the TBP and only the ancestral alleles of the promoters of the *A. thaliana* genes as evaluated in silico and stored in the AtSNP_TATAdb (X-axis) and the log_2_-value of the promoter strength (Y-axis), which were measured in vivo under the experimental conditions described in the caption to this axis on parts (**a**–**f**) of this figure according to article [125]. Solid and dash-and-dot lines denote linear regression and boundaries of its 95% confidence interval, calculated by means of software package STATISTICA version 10.0 (Statsoft^TM^, Tulsa, OK, USA). Statistics: r, R, τ, γ and p are coefficients of Pearson’s linear correlation, Spearman’s rank correlation, Kendall’s rank correlation, Goodman–Kruskal generalized correlation, and their *p*-values (statistical significance), respectively.

Because the most debatable are doubtless the in silico estimates of the K_D_-values of TBP-promoter complexes stored in the AtSNP_TATAdb knowledge base, we tested them using independent experimental data in vivo [125], as shown in Figure 6 and Table S2.

In total, for 50 ancestral alleles of 90 bp promoters before the starts of protein-coding transcripts from the A. thaliana genome, we found the log_2_-values of promoter strength measured in 6 variants of in vivo conditions [125], as shown in Figure 6 (the captions to the Y axes) and in Table S2 (the column headings). Here the readers can see significant correlations between the K_D_-values (AtSNP_TATAdb: X-axis) and the log_2_-values of the promoter strength (Y-axis), which are robust to variations in statistical criteria, genomic environment of promoters, species and growing conditions of tested plants [125]. Taken together, these correlations indicate that the TBP-promoter complex is an autonomous module in the gene expression regulatory machinery [11] and universally explains about 10% of the variation in eukaryotic gene expression in addition to approximately 90% of this variation, which depends on species, tissue and other factors influencing gene activity [24].

### 2.5. Genome-Wide Pattern of Arabidopsis Proximal Promoter SNPs in Comparison with Other Species

This work is the first to conduct a uniform in silico analysis of 871,707 proximal SNPs in promoters of 27,628 *A. thaliana* genes documented in the Ensembl Plant database [31]. As a result, we have predicted that 54,993 SNPs can significantly alter the TBP binding affinity of these promoters along with the gene expression levels and suggested them as candidate SNP markers for 173 plant beneficial features.

First of all, we found that all these SNPs taken together can significantly reduce the affinity of TBP for the gene promoters (Figure 3), which is consistent with both the theoretical genome-wide predictions [126] and the genome-wide data of the 1000 Genomes Project Consortium for binding sites of transcription factors, including TBP, with the human gene promoters [127] as well as with generally accepted ideas about the influence of mutations on gene functions.

Additionally, we observed the only short region 4 bp long around position −30 relative to the transcription start sites of Arabidopsis thaliana genes, where the frequencies of SNPs damaging TBP binding to the promoters are statistically significantly higher than those of SNPs that improve TBP-promoter affinity (Figure 4). This whole-genome result corresponds to both the Bucher criterion [6] for TATA boxes in the promoters of all eukaryotic genes and the predominant localization of TATA boxes in the promoters of plant [13] genes.

At last, for the first time we discovered approximately equal shares of proximal SNPs, which downregulate (3.2%) and upregulate (3.2%) the promoter activity of the protein-coding genes in *Arabidopsis thaliana* (Table 1) along with the mosaic structure of the proximal promoter, enriched predominantly in one or another type of these SNPs (Figure 4). Together with the (A+T)-enrichment of plant promoters, which has been generally recognized for over a quarter of a century [14], this result may fit a balance between the best efficiency of gene functioning, on the one hand, and, on another hand, a necessity of intraspecific biodiversity as a guarantor of survival kept by the stabilizing effect of natural selection.

Summarizing this discussion, we would like to emphasize that, first of all, the influence of SNP on plant gene expression goes far beyond the TBP-promoter binding studied in this work. It may include changes in both the epigenetic status of promoters and the efficiency of their binding to other transcription factors, as well as the density of packaging these promoters into chromatin. So, the annotation we have provided is only a part of an adequate genome-wide systematization of the promoter SNP roles in gene expression.

## 3. Materials and Methods

### 3.1. In Silico Analysis of DNA Sequences

DNA sequences for ancestral and minor alleles of each *Arabidopsis thaliana* gene promoter were retrieved from the Ensembl Plant database [31] using the BioPerl standard library [128]. Each pair of sequences, representing the ancestral and minor alleles, was analyzed in the automatic operating mode of our Web service Plant_SNP_TATA_Z-tester [24], as described in Section S1. The lists of homologous genes of *Arabidopsis thaliana* were taken from the paralogs section of the TAIR database [38].

### 3.2. The Knowledge Base AtSNP_TATAdb Created in this Work

The output data of Plant_SNP_TATA_Z-tester [24] altogether with their hand-curated annotations (Table S1) were written as a textual flat file in an Excel-compatible format. Finally, in the MariaDB 10.2.12 Web environment (MariaDB Corp AB, Espoo, Finland), this flat file was added to our knowledge base AtSNP_TATAdb (https://www.sysbio.ru/AtSNP_TATAdb/), as accessed on 15 December 2023.

### 3.3. How to Use the Knowledge Base AtSNP_TATAdb

We tried to develop a “user-friendly Web-based interface” [129] that could itself be intuitively clear as depicted in Figure 2. First, clicking on the far-left button “Start (Homolog)”, users can work with the knowledge base in the “On-line” mode. Furthermore, we added two buttons: the middle “Start (usual)” button, which by clicking on, anyone can obtain the complete list of 54,993 candidate SNP markers that significantly alter the TBP affinity for the promoters of *A. thaliana* protein-coding genes. Finally, users can download the complete Excel-formatted flat file of the AtSNP_TATAdb knowledge base by clicking on the far-right button “Download DB”.

### 3.4. Statistical Analysis

Statistical analysis in Plant_SNP_TATA_Z-tester [24] was carried out by the standard statistical package R [36], as shown in Figure 2 and Figure 5. To calculate the statistical estimates presented in Table 1 and Table S2 along with Figure 3, Figure 4 and Figure 6 we used the widely accepted toolbox STATISTICA version 10.0 (Statsoft^TM^, Tulsa, OK, USA).

## 4. Conclusions

In this work we identified in silico the 54,993 SNPs able to significantly alter the TBP binding affinity to promoters of *A. thaliana* protein-coding genes for the first time and made these genome-wide finding freely available through our knowledge base AtSNP_TATAdb, https://www.sysbio.ru/AtSNP_TATAdb/.

Additionally, we presented some evidence that these candidate SNP markers can influence plant development and responses to environmental changes. Thus, they may be a part of intraspecific diversity, which is a guarantor of species survival kept by the stabilizing natural selection. Because natural selection is commonly associated with vital traits, we selectively hand-annotated some of the genes carrying these in silico estimated SNPs and found the effects of changes in the gene expression on plant vital traits. We deposited these findings in the knowledge base AtSNP_TATAdb.

Finally, we presented the significant correlations of the in silico estimates of the K_D_-values of TBP-promoter complexes stored in the AtSNP_TATAdb with independent experimental data in vivo [125] that appeared to be robust to variations in statistical criteria, genomic environment of TATA boxes, plant species and growing conditions.

Here we show that SNPs in proximal promoters can change gene expression and suppose that AtSNP_TATAdb can be applied to search for molecular markers of crop advantages in plant breeding.

## Figures and Tables

**Table 1:** A summary of the search for candidate molecular markers of plant advantages related to *Arabidopsis thaliana* proximal promoter SNPs using the Ensembl Plant database [31] along with Plant_SNP_TATA_Z-tester [24], as schematically shown in Figure 1, the result of which is freely available in the AtSNP_TATAdb database created within this work (https://www.sysbio.ru/AtSNP_TATAdb/), as illustrated in Figure 2.

#	Indicator	Result
Genome-wide analysis in silico:		
1	Total number of the *Arabidopsis thaliana* genes documented in the Ensembl Plants database [31] and, thus, taken into account	32,833 (100%)
2	Total number of the *Arabidopsis thaliana* protein-coding genes, which were selected for further analysis	27,628 (80.04%)
3	Total number of the transcripts from the *Arabidopsis thaliana* genes taken into account	54,013 (100%)
4	Total number of the protein-coding transcripts selected from the above amount of the *Arabidopsis thaliana* transcripts, which were selected for further analysis	48,321 (89.46%)
5	Total number of the nucleotide substitutions (SNPs) localized in the proximal region of 90 bp in length just before TSS for the analyzed transcripts	871,707 (100%)
6	Total number of candidate SNP markers having significant changes in the TBP binding affinity identified in this work	54,993 (6.31%)
7	Total number of candidate SNP markers that down-regulated gene expression	27,568 (3.16%)
8	Total number of candidate SNP markers that up-regulated gene expression	27,425 (3.15%)
9	Total number of *A. thaliana* genes whose expression may be significantly altered by the promoter proximal SNPs changing the TBP binding affinity	18,636 (56.78%)
10	The arithmetic mean estimate and its standard error rate of the equilibrium dissociation constant (K_D_) expressed in nanomoles per liter (nM) of the complexes between the TBP and only the ancestral alleles of the promoters of the *Arabidopsis thaliana* genes (Mean ± SEM)	3.96 ± 0.01 nM
11	The arithmetic mean estimate and its standard error rate of the same K_D_-values in the case of only the minor alleles of the *Arabidopsis thaliana* promoters	4.07 ± 0.01 nM
12	Statistical significance of the difference between ancestral and minor alleles of the *Arabidopsis thaliana* promoters examined by the arithmetic mean of the K_D_-values in question according to the following:	
	Student’s *t*-test, *t*-value (P_ADJ_)	8.07 (10^−7^)
	Fisher’s Z-test, Z-score (P_ADJ_)	7.78 (10^−2^)
13	The standard deviation of the same K_D_-values in the case of only the ancestral alleles of the *Arabidopsis thaliana* promoters	1.95 nM
14	The standard deviation of the same K_D_-values in the case of only the minor alleles of the *Arabidopsis thaliana* promoters	1.90 nM
15	Significance of the difference between ancestral and minor alleles of the *A. thaliana* promoters studied by the standard deviation of the K_D_-values by means of Fisher’s F-test and F-score (P_ADJ_)	1.06 (10^−2^)
16	Statistical significance of the difference between ancestral and minor alleles of the *Arabidopsis thaliana* promoters examined as the difference between the distributions of the K_D_-values according to the following:	
	Pearson’s chi-squared test, *χ*^2^-value (P_ADJ_)	298.17 (10^−9^)
	Kolmogorov–Smirnov test, D-score (P_ADJ_)	0.04 (10^−9^)
17	Total number of the *Arabidopsis thaliana* protein-coding genes whose SNP-related expression changes were hand annotated (Supplementary Materials: Table S1)	109
18	Total number of the plant benefits annotated within this work	173
19	Total number of the plant species whose gene expression changes were taken into account within the framework of our hand-curated annotation	17
20	Total number of the original articles on experimental observations of benefits from gene expression alterations cited in this work	83
21	Total number of the proximal promoter SNPs, which can statistically significantly alter expression of the *Arabidopsis thaliana* genes and their paralogs annotated within this work	2426

Notes. P_ADJ_, significance level according to Bonferroni correction for multiple comparisons.

## Data Availability

All original in silico data presented in this work are freely available in the knowledge base AtSNP_TATAdb (https://www.sysbio.ru/AtSNP_TATAdb/) created and presented in this work, as accessed on 15 December 2023.

## Supplementary Materials

The following supporting information can be downloaded at: https://www.mdpi.com/article/10.3390/ijms25010607/s1.

## Abbreviations

ARF1	auxin response factor 1
ARF6	auxin response factor 6
K_D_	equilibrium dissociation constant
nM	natural logarithm units
SNP	single-nucleotide polymorphism
TAIR	*The Arabidopsis Information Resource*
TBP	TATA-binding protein
TSS	transcription start site
