# Supplementary material for: AtSNP_TATAdb: Candidate Molecular Markers of Plant Advantages Related to Single Nucleotide Polymorphisms within Proximal Promoters of Arabidopsis thaliana L
Source: Int J Mol Sci. 2024 Jan 3;25(1):607. doi: 10.3390/ijms25010607 (PMC10779315; doi:10.3390/ijms25010607)
Supplement: Supplementary file 1 [file ijms-25-00607-s001.zip › ijms-2720584-supplementary.pdf]

Supplementary Materials

AtSNP_TATAdb: Candidate Molecular Markers of Plant Advantages Related to Single Nucleotide Polymorphisms within Proximal Promoters of *Arabidopsis thaliana* L.

Anton Bogomolov[1], Karina Zolotareva[1], Sergey Filonov[1,2], Irina Chadaeva[1], Dmitry Rasskazov[1], Ekaterina Sharypova[1], Nikolay Podkolodnyy[1,3], Petr Ponomarenko[1], Ludmila Savinkova[1], Natalya Tverdokhleb[1], Bato Khandaev[1,2], Ekaterina Kondratyuk[1,4], Olga Podkolodnaya[1], Elena Zemlyanskaya[1,2], Nikolay A. Kolchanov[1,2] and Mikhail Ponomarenko[1,*]

[1] Institute of Cytology and Genetics, Novosibirsk 630090, Russia
[2] Natural Science Department, Novosibirsk State University, Novosibirsk 630090, Russia
[3] Institute of Computational Mathematics and Mathematical Geophysics, Novosibirsk 630090, Russia
[4] Siberian Federal Scientific Centre of Agro-BioTechnologies of the Russian Academy of Sciences, Krasnoobsk, Novosibirsk region, Russia
[*] Correspondence: pon@bionet.nsc.ru. Tel.: +7 (383) 363-4963 ext. 1311 (M.P.)

Section S1. Plant_SNP_TATA_Z-tester as a Web service, automatic mode which was used to uniformly estimate how promoter-proximal SNPs can alter *Arabidopsis* gene expression in this work

In this work we used an automatic mode of our previously developed Web-service Plant_SNP_TATA_Z-tester [24] shown in Figures 1 and 5 (hereinafter, see the main text), the input data of which is two DNA sequences 90 bp long located just upstream the transcription start site (TSS, $s^{wt}_0 = s^{min}_0$, where: $s^{\bullet}_i \in \{a, c, g, t\}$) of the proximal promoter analyzed of the *Arabidopsis thaliana* gene under study, such as: $S_{wt} = \{s^{wt}_{-90} \ldots s^{wt}_i \ldots s^{wt}_{-1}\}$ and $S_m = \{s^{min}_{-90} \ldots s^{min}_i \ldots s^{min}_{-1}\}$ corresponding to ancestral (wt) or minor (min) allele, respectively, of a given SNP examined.

The result (i.e., output data) of this analysis is two quantitative sequence-based estimates *in silico* for two "$-\ln[K_{D\,AtTBP}(S_\bullet)]$"-values expressed on the natural-logarithm scale (ln-units), which evaluate the TBP-promoter affinity upon each DNA sequences ($S_\bullet$) independently from one another [24], such as:

$$-\ln[K_{D;\,AtTBP}(S_\bullet)] = 7.0 - 0.6\ln(K_{D;\,HsTBP}(S_\bullet)). \qquad \textbf{(S1)}$$

where 7.0 and 0.6 are linear regression coefficients, which were optimized by us in our previous article [24] using unique data from experimental measurements of the effect of the same mutations in the same promoter on the transcriptional activity of this promoter under the same conditions *ex vivo* using TPB extracted from either *Arabidopsis thaliana* TBP (AtTBP) or human TBP (HsTBP) [32]; $K_{D;\,AtTBP}$ and $K_{D;\,HsTBP}$ are the equilibrium dissociation constant estimation expressed in moles per liter, M, of the TBP extracted from either human or *Arabidopsis thaliana* for the considered alleles (i.e., $S_\bullet$ as S_{wt} or S_m) of the *Arabidopsis thaliana* promoter upon their known DNA sequences of 90 bp in length.

First of all, within Eq. S1, $-\ln(K_{D;\,HsTBP}(S_\bullet))$ is calculated using our three-step model [25] of the TBP-promoter binding (i.e., TBP slides along DNA [26] ↔ molecular co-recognition between TBP and TBP-site met [27] ↔ DNA-bend stabilizes TBP-promoter complex [28]) which is proven experimentally [29], as follows:

$$-\ln[K_{D;\,HsTBP}(S_\bullet)] = 10.9 - 0.2\,\{\ln[K_{SLIDE;\,HsTBP}(S_\bullet)K_{STOP;\,HsTBP}(S_\bullet)K_{BEND;\,HsTBP}(S_\bullet)]\}, \qquad \textbf{(S2)}$$

where; 10.9 (ln-units) seems to numerically match nonspecific TBP–DNA affinity (10 μM) as measured independently [33]; 0.2 is a stoichiometric coefficient of the three-step TBP–promoter binding, as determined elsewhere by means of the difference in the length of the TBP consensus site and the region of TBP sliding along DNA [25].

Besides, within Eq. S2, $-\ln[K_{STOP}(S\bullet)]$ is an estimation of the equilibrium dissociation constant of the mutual recognition between TBP and the most probable TBP-site encountered at the second step among the three steps in question:

$$-\ln[K_{STOP;\ HsTBP}\ (S\bullet)] = MAX_{-90 \leq i \leq -20;\ k \in \{-1;+1\}}\{\Sigma_{i-1 \leq j \leq i+13}\ w\{i,s^{\bullet}_{j;k}\}, \quad \textbf{(S3)}$$

where $w\{i,s^{\bullet}_j\}$ is Bucher's weight of nucleotide $s^{\bullet}_j$ at the *j*th position of the TBP-site [6]; k is an indicator of either a direct (+1) or complementary (–1) strand of the double-stranded B-helical DNA of the promoter under study.; MAX(ζ) is the highest ζ-value observed.

Additionally, in Eq. S2, $-\ln[K_{SLIDE;\ HsTBP}\ (S\bullet)]$ is an estimate of the equilibrium dissociation constant of an interaction between TBP and the promoter DNA during their sliding one over the other at the first step among the three within this bioinformatics model, as:

$$-\ln[K_{SLIDE;\ HsTBP}(S\bullet)] = MEAN_{[\xi-7;\ \xi+19];\ k \in \{-1;+1\}}\ (35.1\mu + 0.8[TA]), \quad \textbf{(S3)}$$

where ξ is the position of the most probable TBP-site according to Bucher's criterion [6] (i.e., Eq. S3); the μ value of the minor-groove width of the B-helical DNA at this site's center was determined elsewhere [34]; [TA] is the concentration of dinucleotide TA; 0.8 and 35.1 are linear regression coefficients [35].

Finally, in Eq. S2, $-\ln[K_{BEND;\ HsTBP}(S\bullet)]$ is an estimation of the equilibrium dissociation constant of intermediate short-lived complexes between TBP and each of two DNA strands of the TBP-site separately from one another during DNA melting leading to the bend that fixes the TBP–promoter complex [28] at the last step of their binding, as follows:

$$-\ln[K_{BEND;\ HsTBP}(S\bullet)] = MEAN_{[\xi-7;\xi+19];\ k \in \{-1;+1\}}\ (0.9[TA, AA, TG, AG] + 2.5[TA, TC, TG] + 14.4), \quad \textbf{(S4)}$$

where 0.9, 2.5, and 14.4 are linear regression coefficients [35].

After that, examining all the possible mutations, $s^{\bullet}_j \rightarrow \varphi$, at each *j*th position among 26 positions of the most probable TBP-site according to Eq. S3, our automatic mode of Web-service Plant_SNP_TATA_Z-tester [24] estimated standard error of the mean SEM● of the $-\ln[K_{D;\ AtTBP}\ (S\bullet)]$ values calculated using Eq. S1, as:

$$SEM_{\bullet}=\{(\Sigma_{\xi-7 \leq j \leq \xi+19}\Sigma_{\varphi\ \{a,c,g,t\}}\ln[K_{D;\ AtTBP}\ (s^{\bullet}_{\xi-7}\ldots s_{j-1}\varphi s_{j+1}\ldots s^{\bullet}_{\xi+19}) / K_{D;\ AtTBP}\ (s^{\bullet}_{\xi-7}\ldots s_{j-1}s_j\ s_{j+1}\ldots s^{\bullet}_{\xi+19})]^2\} / ((3*26)(3*26-1))]^{1/2}. \quad \textbf{(S6)}$$

Using both sequences S_{wt} and S_{min} and Eqs. S1 - S5, this toolbox found two paired value sets $\{-\ln[K_D(S_{wt})] \pm SEM_{wt}\}$ and $\{-\ln(K_{D;\ AtTBP}\ (S_{min})) \pm SEM_{min}\}$, respectively, which are necessary for Fisher's Z-score [36], for instance:

$$Z = abs\{\ln[K_{D;\ AtTBP}\ (S_{wt}) / K_{D;\ AtTBP}\ (S_m))] / [SEM_{wt}^2 + SEM_m^2\}^{1/2}.. \quad \textbf{(S7)}$$

Eventually, with the help of the R software [36], using this Z-value, our Web-service Plant_SNP_TATA_Z-tester [24] found a *p* value of the probability of the tested hypothesis "H_0: $K_{D;\ AtTBP}\ (S_{wt}) \neq K_{D;\ AtTBP}\ (S_{min})$" so that if it is statistical significant ($p > 0.95$), it made the decision:

IF {**INEQUALITY** "$K_{D;\ AtTBP}\ (S_{min}) < K_{D;\ AtTBP}\ (S_{wt})$" is statistically significant },

THEN {**DECISION** is "the minor allele of the gene considered is overexpressed relative to the ancestral one"};

ELSE [**IF** {**INEQUALITY** "$K_{D;\ AtTBP}\ (S_m) > K_{D;\ AtTBP}\ (S_{wt})$" is statistically significant}, **(S8)**

THEN {**DECISION** is "the minor allele of this gene is underexpressed relative to the ancestral one"},]

OTHERWISE {**DECISION** is "the expression change of this gene is insignificant"}.

Dialogue mode of our Web service Plant_SNP_TATA_Z-tester [24] presents this decision (Eq. S8) in the "Decision" line of the "Result" textbox, while all the intermediate results are in the other lines of this textbox, as readers can see in Figure 5.

Table S1. Candidate SNP markers within the 90-bp proximal promoters of the *Arabidopsis thaliana* genes taken as an example of filling out the AtSNP_TATAdb database, created for the first time in this work.

#	*A. thaliana* gene (Entrez ID [37]; TAIR ID [38])	Candidate SNP marker				KD, nM, MEAN ± SEM, *in silico*					Hand-curated annotation using the PubMed database [39]		
		TAIR SNP ID [38]): minor allele	5′ flank, 10 bp	WT<br>min	3 flank, 10 bp	WT<br>min	Δ	Z	p	ρ	economically valuable traits	Effect on this trait (ϒ: "∧" as improved; "∨" as impaired) within plant breeding models using *Arabidopsis thaliana* [Ref]	ϒ
1	*ARF1* (842268; AT1G59750)	ENSVATH01403825:A	taaaaaaaaa	T<br>A	ataaagagga	2.84±0.18<br>5.29±0.24	↓	16.29	10^{-6}	A	somatic embryogenic response	within plant somatic embryogenesis models using *Arabidopsis thaliana* (ecotype Columbia) treated with auxin and, next, tested with qPCR: ARF1 deficit is a molecular marker for somatic embryogenesis as well as mutational ARF1-downregulation can impair somatic embryogenic response [40]	∨
		ENSVATH01403824:T	aattaaaaaaa	A<br>T	atataaagag	2.84±0.18<br>1.65±0.11	↑	11.94	10^{-6}	A	leaf senescence, chlorophyll content	within plant development models using *Cymbidium goeringii* as an economically important genus of flowering orchids cultivated in China: ARF1 overexpression in leaves is a molecular marker for both leaf senescence and decreased chlorophyll content [41]	∨
2	*ARF3* (817014; AT2G24765)	ENSVATH05582399:A	gagctgataa	T<br>A	atttaggccc	4.05±0.27<br>6.11±0.32	↓	9.77	10^{-6}	A	somatic embryogenic response	within plant somatic embryogenesis models using *Arabidopsis thaliana* (ecotype Columbia) treated with auxin and, next, tested with qPCR: ARF3 deficit is a molecular marker for somatic embryogenesis as well as mutational ARF1-downregulation can impair somatic embryogenic response [40]	∨
3	*ARF6* (839913; AT1G30330)	tmp_1_10691073_T_A:T	accataacgt	A<br>T	ttattaaaac	3.34±0.19<br>3.65±0.19	↓	2.27	0.05	D	lower buds opening	within plant development models using arf6 arf8 double loss-of-function mutant Arabidopsis lines: arrested flowers just before wild-type flower buds normally open [42]	∨
		ENSVATH04674378:A	gggttcgggt	C<br>A	aaaaaacagc	7.94±0.38<br>5.36±0.29	↑	10.91	10^{-6}	A	somatic embryogenic response	within plant somatic embryogenesis models using *Arabidopsis thaliana* (ecotype Columbia) treated with auxin and, next, tested with qPCR: ARF6 excess is a molecular marker for somatic embryogenesis [40]	∧
		ENSVATH12375115:A	tgctttagaa	G<br>A	aaaatcaaag	7.94±0.38<br>6.86±0.35		4.20	10^{-3}	B			
4	*ARF8* (833672; AT5G37020)	ENSVATH14527658:T	tgacacatat	C<br>T	tttttctctc	5.70±0.34<br>6.22±0.37	↓	2.08	0.05	D	lower buds opening	within plant development models using arf6 arf8 double loss-of-function mutant Arabidopsis lines: arrested flowers just before wild-type flower buds normally open [42]	∨
		tmp_5_14629589_T_A:A	ctctgacac	T<br>A	atcttttttct	5.70±0.34<br>11.21±0.52		17.93	10^{-6}	A			
		ENSVATH12056628:A	aagtgactct	G<br>A	gtactgacac	5.96±0.52<br>4.42±0.32	↑	5.28	10^{-6}	A	somatic embryogenic response	within plant somatic embryogenesis models using Arabidopsis thaliana (ecotype Columbia) treated with auxin and, next, tested with qPCR: ARF8 excess is a molecular marker for somatic embryogenesis [40]	∧
		tmp_5_14629402_C_A:A	caccacctct	C<br>A	ataattctcg	5.96±0.52<br>4.49±0.29		5.26	10^{-6}	A			
5	*ARF10* (817382; AT2G28350)	ENSVATH13446932:A	ctcttcttct	T<br>A	ctacgatctg	4.86±0.35<br>3.70±0.23	↑	5.75	10^{-6}	A	somatic embryogenic response	within plant somatic embryogenesis models using *Arabidopsis thaliana* (ecotype Columbia) treated with auxin and, next, tested with qPCR: ARF10 excess is a molecular marker for somatic embryogenesis [40]	∧
		tmp_2_12113993_C_T:T	ctgattatag	C<br>T	atctctctct	4.86±0.35<br>2.81±0.21		10.56	10^{-6}	A			

Note: *Alleles*: WT, ancestral; min, minor; K_D, dissociation constant of the TBP–promoter complex; Z and p, Fisher's Z-test and its statistical significance estimate; ρ, heuristic rank of candidate SNP markers from the "best" (A) to the "worst" (E); Δ, expression change: "↓" as deficit, "↑" as excess

Table S1. *Cont.*

#	*A. thaliana* gene (Entrez ID [37]; TAIR ID [38])	Candidate SNP marker				K_D, nM, MEAN ± SEM, *in silico*					Hand-curated annotation using the PubMed database [39]		
		TAIR SNP ID [38]): minor allele	5′ flank, 10 bp	WT min	3 flank, 10 bp	WT min	Δ	Z	p	ϱ	economically valuable traits	Effect on this trait (ϒ: "∧" as improved; "∨" as impaired) within plant breeding models using *Arabidopsis thaliana* [Ref]	ϒ
6	*ARF11* (819264; AT2G46530	tmp_2_19104613_T_C:C	acacgtagtt	T<br>C	gaaagatgag	4.64±0.26<br>6.82±0.35	↓	10.12	10^{-6}	A	somatic embryogenic response	within plant somatic embryogenesis models using Arabidopsis thaliana (ecotype Columbia) treated with auxin and, next, tested with qPCR: the activity of ARF11 transcripts were four-times less abundant in the somatic embryos-induced explants than in the freshly isolated explants as the most reduced in embryogenesis [40]	∨
		ENSVATH05729071:A	agtcaacatt	T<br>A	ttaactctaa	4.93±0.23<br>4.59±0.25	↑	1.98	0.05	D	fast-growing plants	within plant development models using poplar as an important fast-growing tree, transcriptomes of which have been profiled for several tissues: ARF11 overexpression in the 10th leaf position may be a molecular marker for fast-growing plants [43]	∧
		tmp_2_19105101_T_A:A	tcaacatttt	T<br>A	aactctaatc	4.93±0.23<br>4.10±0.21		5.34	10^{-6}	A			
7	*ARF12* (840331; AT1G34310)	tmp_1_12511576_C_T:A	agagaaagtt	G<br>A	tataataatt	2.67±0.17<br>2.10±0.13	↑	5.26	10^{-6}	A	root shape, root thickness, root depth	within plant development models using moroberekan with deep and thick roots and high root/shoot ratio in comparison with others rice lines either with numerous but shallow roots or with fewer but deeper roots than the latter ones: ARF12 excess may be a molecular marker for deep roots under drought, thick and deep roots under low nitrogen content as well as new thick and deep roots after trimming [44]	∧
9	*ARF17* (844120; AT1G77850)	ENSVATH01551471:G	attcagtgag	T<br>G	tttaataaca	3.14±0.20<br>3.49±0.27		2.12	0.05	D	male sterility, male subfertility	within plant fertility models using ARF17 loss-of-function *Arabidopsis thaliana* mutant (ecotype Columbia): male sterility [45]	∨
		ENSVATH05163913:C	gagagatatt	A<br>C	aaagtttgtt	3.38±0.18<br>3.96±0.25	↓	3.80	10^{-3}	B			
		ENSVATH05163913:T	gagagatatt	A<br>T	aaagtttgtt	3.38±0.18<br>3.74±0.23		2.49	0.05	D			
		ENSVATH01551472:T	gttttaataa	C<br>T	aataataaaa	3.14±0.20<br>2.78±0.17	↑	2.80	10^{-2}	C		in plant fertility models using *A. thaliana* (ecotype Columbia): ARF17 excess is a molecular marker for male sterility or subfertility [46]	∨
10	*ARF18* (825356; AT3G61830)	ENSVATH00431478:G	gtgagtagta	A<br>G	aaggctgaga	3.39±0.20<br>4.61±0.32	↓	6.76	10^{-6}	A	shade response, stem growth, leaf area	within plant shade avoidance models using ARF18-knockout *Arabidopsis thaliana* (ecotype Columbia): enhances auxin-dependent hypocotyl elongation accelerating stem growth and narrowing leaf area [47]	∧
		ENSVATH06357481:T	aaaaaaaaaa	G<br>T	attttaagag	6.85±0.34<br>5.14±0.22	↑	8.75	10^{-6}	A		within plant shade avoidance models using ARF18-knockin *Arabidopsis thaliana* (ecotype Columbia): reduced shade responsiveness, shorted hypocotyl slowing down stem growth and expanding leaf area [47]	∨
11	ARF19 (838505; AT1G19220)	tmp_1_6633212_T_A:T	aaaaagaaat	A<br>T	aaaaaggtgg	4.86±0.26<br>5.79±0.25	↓	5.15	10^{-6}	A	lateral root formation	within plant development models using doubleARF7\ARF19-knockout Arabidopsis thaliana (ecotype Columbia): severely impaired in lateral root formation [48]	∨
		ENSVATH04513629:A	taaaaagaaa	T<br>A	aaaaaaggtg	4.86±0.26<br>6.19±0.36		6.19	10^{-6}	A			
		ENSVATH11518153:T	aagaaataaa	A<br>T	aaggtgggac	4.86±0.26<br>4.06±0.22	↑	4.83	10^{-3}	B	ethylene response, auxin response, phytohormones cross-talk	in plant phytohormone response models using *Arabidopsis thaliana* (ecotype Columbia) treated with either auxin or ethylene: ARF19 excess is a molecular marker of response to both auxin and ethylene as a cross-talk between these phytohormones complementing each other [49]	∧
		tmp_1_6633217_C_A:T	aaaataaaaa	G<br>T	aaataaaaaa	4.86±0.26<br>4.35±0.22		2.99	10^{-2}	C			

Table S1. *Cont.*

#	*A. thaliana* gene (Entrez ID [37]; TAIR ID [38])	Candidate SNP marker				K_D, nM, MEAN ± SEM, *in silico*					Hand-curated annotation using the PubMed database [39]		
		TAIR SNP ID [38]): minor allele	5′ flank, 10 bp	WT<br>min	3 flank, 10 bp	WT<br>min	Δ	Z	p	ϱ	economically valuable traits	Effect on this trait (ϒ: "∧" as improved; "∨" as impaired) within plant breeding models using *Arabidopsis thaliana* [Ref]	ϒ
12	ARF20 (840413; AT1G35240)	ENSVATH14089833:A	gagaaagttt	T<br>A	ataataattg	2.88±0.17<br>3.93±0.21	↓	7.83	10^{-6}	A	bacterial effect, auxin accumulation response, root architecture, root growth, seedlings development, lateral roots, roots hair, root density	within models of how bacteria can effect to plant root development using Arabidopsis thaliana: *Lysinibacillus macroides* downregulated ARF20 in seedlings that coincides with improved root both growth and architecture by means of increased numbers of lateral roots, roots hair, root density as a response to auxin accumulation [50]	∧
		tmp_1_12930582_C_A:T	aagggagaaa	G<br>T	ttttataata	2.88±0.17<br>2.57±0.15	↑	2.71	10^{-2}	C		as a heuristic contrast to the only ARF20-related publication found about the model of how bacteria can effect to plant root development using Arabidopsis thaliana, when Lysinibacillus macroides downregulated ARF20, ARF20 upregulation might somehow impair seedlings root development via defective root either growth or architecture by means of decreased numbers of lateral roots, roots hair, root density [50]	∨
13	ARF22 (840341; AT1G34390)	tmp_1_12555954_A_C:C	aagttgtata	A<br>C	taattgttgg	2.67±0.15<br>3.07±0.24	↓	2.94	10^{-2}	C	nitrogen deficiency stress	within models of plant stress response using *Triticum turgidum subsp. Durum*: ARF20 downregulation is a molecular marker in roots under both chronic and short-term nitrogen deficiency stress [51]	∧
		ENSVATH12859519:C	gtataataat	T<br>C	gttgggataa	2.67±0.15<br>2.40±0.16	↑	2.38	0.05	D	inorganic phosphate deficiency stress response	within models of plant stress response using *Camellia oleifera*: *ARF20* upregulation is a hub molecular marker for inorganic phosphate deficiency stress response [52]	∧
14	GH3.9 (819387; AT2G47750)	tmp_2_19563976_T_G:C	acatttaaaa	A<br>C	cccacatagg	3.55±0.20<br>4.14±0.30	↓	3.36	10^{-3}	B	auxin response, seedlings growth, root growth, root length	within plant development models using transgenic, additional Arabidopsis thaliana with reduced GH3.9 expression due to RNA interference: a longer primary root length and increased sensitivity to auxin-mediated inhibition of root growth in seedlings [53]	∧
15	IAA5 (838128; AT1G15580)	ENSVATH04582086:C	catgagaaca	T<br>C	ataaagtggc	2.86±0.22<br>4.60±0.30	↓	9.47	10^{-6}	A	gravitropism, gravitropic response indicator	within plant gravitropism models using Arabidopsis thaliana: IAA5 expression level decreases from root to inflorescence stems as a quantitative gravitropic response indicator [54]	∧
16	IAA6 (841717; AT1G52830)	ENSVATH01359837:T	gacaacatat	A<br>T	aaagcacgag	2.31±0.14<br>2.61±0.21	↓	2.48	0.05	D	adventitious root initiation	within plant development models using IAA6 loss-of-function mutant *Arabidopsis thaliana*: IAA6-deficit may be a molecular marker of root under light as an additive controller for initiation of adventitious root [55]	∧
17	IAA8 (816798; AT2G22670)	ENSVATH13333330:G	agatctaacta	C<br>G	agtactccac	4.95±0.34<br>5.67±0.38	↓	2.81	10^{-2}	C	lateral root initiation	within plant development models using IAA8 loss-of-function mutant *Arabidopsis thaliana*: significantly more lateral roots [56]	∧
		ENSVATH01867835:T	cgtcagatct	A<br>T	actacagtac	4.95±0.34<br>4.24±0.31	↑	3.09	10^{-2}	C	lateral root initiation, gravitropism	within plant development models using transgenic Arabidopsis thaliana carrying an additional estrogen-inducible IAA8 gene copy: significantly fewer lateral roots and abnormal gravitropism [56]	∨
		ENSVATH05558337:A	ccttactttt	G<br>A	tagtttatta	3.36±0.21<br>1.78±0.11		14.22	10^{-6}	A			

Table S1. *Cont.*

#	*A. thaliana* gene (Entrez ID [37]; TAIR ID [38])	Candidate SNP marker				K_D, nM, MEAN ± SEM, *in silico*					Hand-curated annotation using the PubMed database [39]		
		TAIR SNP ID [38]): minor allele	5′ flank, 10 bp	WT<br>min	3 flank, 10 bp	WT<br>min	Δ	Z	p	ϱ	economically valuable traits	Effect on this trait (ϒ: "∧" as improved; "∨" as impaired) within plant breeding models using *Arabidopsis thaliana* [Ref]	ϒ
18	IAA9 (836693; AT5G65670)	ENSVATH03479832:G	ttagtcatta	A<br>G	aaacaaaagg	5.53±0.24<br>6.60±0.32	↓	5.44	10^{-6}	A	parthenocarpic fruits without fertilization	within models of heuristic artificial selection of plants for economically valuable traits using SlIAA9-knockout tomato (*Solanum lycopersicum*): developed parthenocarpic fruits without fertilization [57]	∧
		ENSVATH03479833:G	tagtcattaa	A<br>G	aacaaaagga	5.53±0.24<br>6.60±0.32	↓	5.47	10^{-6}	A			
		ENSVATH12943606:C	agtcattaaa	A<br>C	acaaaaggat	5.53±0.24<br>5.67±0.38	↓	3.09	10^{-2}	C			
		ENSVATH03479830:T	atagaggtta	C<br>T	attagtcatt	5.53±0.24<br>3.41±0.27	↑	10.53	10^{-6}	A	auxin response, plant growth, begins to flower	within plant growth models using grapevine (*Vitis vinifera L.*) treated with exogenous auxin: VvIAA9 overexpression along with increased berry diameter rapidly, whereas *Arabidopsis thaliana* initially overexpressing AtIAA9 grows quickly and fast begins to flower, but demonstrates hyposensitivity to exogenous auxin [58]	∧
		ENSVATH03479831:G	tagaggttac	A<br>G	ttagtcatta	5.53±0.24<br>5.07±0.23	↑	2.76	10^{-2}	C			
		ENSVATH12943604:A	ctttttctct	C<br>A	tttaccattt	5.53±0.24<br>4.73±0.30	↑	4.05	10^{-3}	B			
		tmp_5_26253337_C_T:T	ctctctttac	C<br>T	attttaccct	5.53±0.24<br>4.27±0.25	↑	7.17	10^{-6}	A			
		tmp_5_26253399_A_G:G	gtcattaaaa	A<br>G	caaaaggata	5.53±0.24<br>5.21±0.22	↑	1.98	0.05	D			
19	IAA10 (839290; AT1G04100)	tmp_1_1059441_A_G:G	ataaagttat	A<br>G	tagacgaaca	2.02±0.17<br>2.70±0.19	↓	5.25	10^{-6}	A	antiviral response	within models of plant antiviral response using rice (*Oryza sativa L.*) infected with rice dwarf virus and, next, treated with exogenous auxin: OsIAA10 downregulated, thereby unleashing antiviral response gene expression [59]	∧
20	IAA11 (828982; AT4G28640)	ENSVATH12279442:A	attattggat	C<br>A	aaaaaaatga	5.73±0.29<br>4.04±0.23	↑	9.21	10^{-6}	A	sucrose response, indole-3-butyric acid response, stem-base tissues, reactive oxygen species scavenging	within models of plant agricultural treatment response using *Carica papaya* plantlets subjected with both sucrose and exogenous indole-3-butyric acid simultaneously: CpIAA11 upregulated in stem-base tissues as an indicator for reactive oxygen species formation or accumulation [60]	∧
		ENSVATH12279443:C	aatgataaaa	T<br>C	tgggcgagaa	5.73±0.29<br>5.06±0.28	↑	3.30	10^{-3}	B			
21	IAA12 (839495; AT1G04550)	tmp_1_1240258_T_C:C	tttgattatg	T<br>C	cggaagacaa	6.85±0.32<br>9.55±0.39	↓	10.71	10^{-6}	A	photo-oxidative stress response, high light	within models of plant light response using *Arabidopsis thaliana*: suppressed IAA12 may be a molecular marker for plant response to photo-oxidative stress caused by excess high light conditions[61]	∧
22	IAA13 (817894; AT2G33310)	ENSVATH05649388:C	cacctaatc	T<br>C	ctctctcttt	10.06±0.50<br>11.13±0.55	↓	2.90	10^{-2}	C	photo-oxidative stress response, high light condition	within models of plant light response using *Arabidopsis thaliana*: suppressed IAA13 may be a molecular marker for plant response to photo-oxidative stress caused by excess high light conditions[61]	∧
		ENSVATH05649389:G	ctccaccacc	T<br>G	aatcctctct	10.06±0.50<br>12.78±0.39	↓	8.18	10^{-6}	A			
		tmp_2_14116146_T_A:T	ttaataataa	A<br>T	attagggaat	3.27±0.15<br>3.58±0.22	↓	2.42	0.05	D			
		tmp_2_14115982_G_A:T	tttcagattt	C<br>T	aagaaaccct	10.06±0.50<br>7.74±0.35	↑	7.79	10^{-6}	A	sulphur starvation response, cysteine excess, dwarf plant	within models of plant sulphur starvation response using Arabidopsis thaliana: IAA13 upregulation along with an increase in cysteine level by 3.5 and the appearance of dwarf plants [62]	∧

Table S1. *Cont.*

#	*A. thaliana* gene (Entrez ID [37]; TAIR ID [38])	Candidate SNP marker				K_D, nM, MEAN ± SEM, *in silico*					Hand-curated annotation using the PubMed database [39]		
		TAIR SNP ID [38]): minor allele	5′ flank, 10 bp	WT min	3 flank, 10 bp	WT min	Δ	Z	p	ϱ	economically valuable traits	Effect on this trait (ϒ: "∧" as improved; "∨" as impaired) within plant breeding models using *Arabidopsis thaliana* [Ref]	ϒ
23	IAA18 (841623; AT1G51950)	tmp_1_19305020_T_A:A	aaatatgttt	T<br>A	aaataaaaag	3.20±0.19<br>3.63±0.20	↓	3.09	10^{-2}	C	host-plant response to viral infection	within models of host-plant response to viral infection using *Arabidopsis thaliana* (ecotype Shahdara) infected with *Tobacco mosaic virus*: IAA18 downregulation is a molecular marker for plant response to *Tobacco mosaic virus* infection [63]	∨
24	IAA19 (820793; AT3G15540)	ENSVATH10697478:A	gaccaacgaa	G<br>A	caacatataa	5.00±0.26<br>3.68±0.18	↑	8.54	10^{-6}	A	plant growth, root elongation, floral transition	within plant growth models using transgenic *Arabidopsis plants* overexpressing *VvIAA19* taken from grapevine (*Vitis vinifera* L.): faster growth, accelerated both root elongation and floral transition without any detectable harm [64]	∧
25	IAA29 (829361; AT4G32280)	ENSVATH02950386:T	acatatatat	G<br>T	tatatttttg	1.55±0.09<br>1.71±0.11	↓	2.25	0.05	D	plant response to water-deficit stress	within models of plant response to water-deficit stress using peanut subjected with water deficit stress: IAA29 downregulation may be a molecular marker for plant response to water-deficit stress [65]	∧
26	IAA30 (825383; AT3G62100)	ENSVATH12879768:G	tccattctct	A<br>G	tatataatac	1.39±0.13<br>1.62±0.12	↓	2.57	0.05	D	plant apomixis	according to the identification of this gene for the first time in *Paspalum notatum* via searching for homology to the rice (*Oryza sativa* L.) gene *OsIAA30* through the *Paspalum notatum* transcriptome: *PnIAA30* downregulated in reproductive tissues of apomictic *Paspalum notatum*, namely: in apomictic spikelets compared to sexual ones [66]	∨
		ENSVATH06359108:T	acatctttaa	C<br>T	aacactgtct	4.82±0.19<br>4.05±0.17	↑	6.08	10^{-6}	A	plant growth, primary root growth, root apical meristem, plant response to high salt stress	within plant growth models using *Arabidopsis thaliana* mutant overexpressing *IAA30*: dwarfed growth with stunted primary root growth because of disturbed root apical meristem activity and seed germination repressed as a response to high salt stress [67]	∧
		ENSVATH06359109:T	ttaattaaaa	A<br>T	aaaaacatat	4.82±0.19<br>4.23±0.18		4.62	10^{-3}	A			
27	IAA34 (838070; AT1G15050)	ENSVATH11247661:C	agctgcttga	T<br>C	ataaatcaac	2.58±0.17<br>3.45±0.27	↓	5.67	10^{-6}	A	apical-hook defect, seedling development start	within plant growth models using double *IAA32-IAA34*-knockout *Arabidopsis thaliana* mutant in comparison with both wild-typed and single either *IAA32* or *IAA34* knockouts: an apical-hook maintenance defects during seedling development starts [68]	∨
		tmp_1_5183068_T_A:T	gcttgatata	A<br>T	atcaacatca	2.58±0.17<br>1.78±0.18	↑	6.11	10^{-6}	A	cell growth, apical-hook formation, seedling development start	within plant growth models using transgenic *Arabidopsis thaliana* carrying IAA34-promoter-driven β-glucuronidase reporter-gene construct: IAA34 upregulation is a molecular marker for reduced cell growth and apical hook formation during seedling development starts [68]	∧
28	SAUR23 (831310; AT5G18060)	ENSVATH03072882:A	ctcatttcat	G<br>A	attaggcttt	4.61±0.27<br>4.20±0.28	↑	2.15	0.05	D	palisade and spongy tissue misbalance, leaves rolling, plant growth, plant development	within plant leaves development models using birch (*Betula pendula*) mutants: BpSAUR23 upregulation along with palisade and spongy tissue misbalance leading leaves rolling as defects in plant growth and development [69]	∨

Table S1. *Cont.*

#	*A. thaliana* gene (Entrez ID [37]; TAIR ID [38])	Candidate SNP marker				K_D, nM, MEAN ± SEM, *in silico*					Hand-curated annotation using the PubMed database [39]		
		TAIR SNP ID [38]): minor allele	5′ flank, 10 bp	WT min	3 flank, 10 bp	WT min	Δ	Z	p	ϱ	economically valuable traits	Effect on this trait (ϒ: "∧" as improved; "∨" as impaired) within plant breeding models using *Arabidopsis thaliana* [Ref]	ϒ
29	SAUR24 (28721172; AT5G18080)	tmp_5_5983636_A_T:T	ctgttttagt	A T	tgtccaacgc	3.39±0.23 6.99±0.36	↓	17.13	10^{-6}	A	hypocotyl length, leaf size, plant growth, plant development	within plant leaves development models using *Arabidopsis thaliana* (ecotype Columbia) treated with exogenous artificial microRNA targeted to SAUR19-24: SAUR24 downregulation along with decrease in the hypocotyl length and leaf size as defects in plant growth and development [70]	∨
30	SAUR26 (821111; AT3G03850)	ENSVATH02109247:A	cacactttta	G A	acaccattgt	5.20±0.29 3.63±0.19	↑	9.35	10^{-6}	A	thermo-responsiveness	within plant thermo-responsiveness models using SAUR26-knockin *Arabidopsis thaliana* (ecotype Columbia): SAUR26-overexpression along with enhanced thermo-responsiveness [71]	∧
31	SAUR27 (821121; AT3G03840)	tmp_3_981048_A_T:T	tattgtatta	A T	tattttcctg	3.00±0.13 3.37±0.17	↓	3.44	10^{-3}	B	thermo-responsiveness	within models of plant adaptation to the environment using polymorphic variants of *Arabidopsis* thaliana (ecotype Columbia) that differ from each other in thermos-responsiveness: plants with higher thermos-responsiveness often have higher expression of SAUR27 and *vice versa* [71]	∨
		ENSVATH07951739:G	tgtattaata	T G	tttcctgatt	3.00±0.13 2.75±0.12		2.74	10^{-2}	C			
		tmp_3_981039_A_C:C	tatttctttt	A C	ttgtattaat	3.00±0.13 2.63±0.14	↑	3.88	10^{-3}	B			∧
32	SAUR68 (839828; AT1G29510)	ENSVATH04665559:T	ctcttgtcat	A T	taaacgtccg	2.83±0.18 3.72±0.20	↓	6.50	10^{-6}	A	heterosis, auxin–induced plant growth	within plant heterosis models using hybrids of Easter lily (*Lilium longiflorum*): LlSAUR68 down–regulation along with enhanced auxin–induced growth in hypocotyls, inflorescence, stems, petals and stamen filaments [72]	∧
		ENSVATH01123843:T	gtcatataaa	C T	gtccgtatag	2.83±0.18 2.24±0.15	↑	5.11	10^{-6}	A	plant growth, hypocotyls growth, petals growth, stamen filaments growth	within plant growth models using transgenic *Arabidopsis thaliana* carrying SAUR68/β-glucuronidase fused reporter-gene construct: SAUR68 gain-of-function along with more longer hypocotyls, petals and stamen filaments [73]	∧
33	SAUR75 (832840; AT5G27780)	ENSVATH03147590:T	atatgttgtt	G T	aaacatatgt	3.57±0.27 4.19±0.25	↓	3.33	10^{-3}	B	fertilization defects, seed abortion, shorter siliques, fewer seeds per silique	within plant fertilization models using homozygous *SAUR75*-knockout *Arabidopsis thaliana*: SAUR75 deficiency along with to fertilization defects such as seed abortion, shorter siliques, and fewer seeds per silique [74]	∨
		tmp_5_9839519_T_G:C	actaaacaaa	A C	tatatgttgt	3.57±0.27 3.06±0.21	↑	3.04	10^{-2}	V	circadian rhythm	within plant circadian rhythm models using *Arabidopsis thaliana*: SAUR75 expression exhibits a circadian rhythm with highest levels of expression in the morning [73]	∧
34	PIN1 (843693; AT1G73590)	ENSVATH05139219:T	aaaaaaataa	A T	ataaaaagaa	2.25±0.15 2.06±0.13	↑	2.00	0.05	D	plant development, root architecture, lateral root primordia,	within plant developmental models using *Arabidopsis thaliana*: in roots, PIN1 excess can lead excessive auxin accumulation in the endodermal, cortical, and epidermal cells surrounding lateral root primordia that can reduced lateral root density [75]	∨

lateral root density

Table S1. *Cont.*

#	*A. thaliana* gene (Entrez ID [37]; TAIR ID [38])	Candidate SNP marker: TAIR SNP ID [38]): minor allele	Candidate SNP marker: 5′ flank, 10 bp	Candidate SNP marker: WT min	Candidate SNP marker: 3 flank, 10 bp	K_D, nM, MEAN ± SEM, *in silico*: WT min	Δ	Z	p	ϱ	Hand-curated annotation using the PubMed database [39]: economically valuable traits	Hand-curated annotation using the PubMed database [39]: Effect on this trait (ϓ: "Λ" as improved; "V" as impaired) within plant breeding models using *Arabidopsis thaliana* [Ref]	ϓ
35	PIN3 (843432, AT1G70940)	ENSVATH01518899:T	ttattttttt	G T	ataagatcga	3.68±0.24 2.76±0.18		6.29	10^{-6}	A	plant development, late ovule initiation, pistil size	within plant ovule initiation models using *Arabidopsis thaliana* mutants: brassinosteroids upregulate *PIN3* that contribute to both late ovule initiation and increased pistil size [76]	Λ
		ENSVATH13788778:T	ttatttaata	A T	ttaaacaaaa	3.68±0.24 2.96±0.20	↑	4.70	10^{-3}	B			
		tmp_1_26742916_T_A:A	agaactatat	T A	gttatttttt	3.68±0.24 2.14±0.20		9.60	10^{-6}	A			
36	PIN4 (814670; AT2G01420)	ENSVATH05203899:A	tttgaaaagg	G A	aataaatgtg	4.66±0.25 5.04±0.27	↓	2.06	0.05	D	plant response to water-deficit stress	within models of plant response to water-deficit stress using *Arabidopsis thaliana* subjected with polyethyleneglycol exposure: *PIN4* downregulation is a molecular marker for plant response to water-deficit stress [77]	Λ
		ENSVATH10501410:A	agtacgtaga	C A	aatagtttga	4.71±0.30 3.53±0.21		6.62	10^{-6}	A	plant development, high-nitrate conditions, shoot-branching	within models of plant response to high-nitrate conditions using *Arabidopsis thaliana*: PIN4 overaccumulation along with increased shoot-branching [78]	Λ
		ENSVATH10501411:A	gtggctgtct	T A	cttgtaccaa	4.71±0.30 3.66±0.23	↑	5.62	10^{-6}	A			
		tmp_2_183708_G_A:T	cttcttgtac	C T	aagtacgtag	4.71±0.30 2.93±0.18		10.74	10^{-6}	A			
37	PIN5 (831515; AT5G16530)	ENSVATH10785668:C	tgtttaattt	A C	tatctaaac	3.77±0.19 4.32±0.22	↓	3.79	10^{-3}	B	root meristem size, root growth	within plant developmental models using PIN5-deficient *Arabidopsis thaliana*: enlarged root meristem size and enhanced root growth [79]	Λ
		tmp_5_5400527_C_T:T	tatatctaaa	G T	tttacttttg	3.77±0.19 4.35±0.25		3.69	10^{-6}	A			
38	PIN7 (838916; AT1G23080)	ENSVATH01091865:G	ctaagatata	T G	atagtattaa	1.36±0.08 2.06±0.14	↓	9.37	10^{-6}	A	gravitropism, lateral root bending	within plant gravitropism models using PIN7-deficient Arabidopsis thaliana: lateral roots display upward bending instead of downward bending as impaired gravitropism [80]	V
		ENSVATH01091864:G	atatatagta	T G	taaaaagtac	1.45±0.10 1.27±0.07	↑	3.07	10^{-2}	C	rhizobacteria beneficial effect, plant development, gravitropism	within models of the beneficial effect of rhizobacteria on plant development using *Arabidopsis thaliana* subjected with roots inoculation by *Bradyrhizobium japonicum*: host plant transcriptional reprogramming to overexpress PIN7 resulting in improved gravitropism [81]	Λ
		ENSVATH01091866:C	ggggaaagct	A C	agatatatat	1.36±0.08 1.18±0.09		3.07	10^{-2}	C			
39	PIN8 (831362; AT5G15100)	tmp_5_4894180_G_T:A	ataaattaaa	C A	ccagtgtttt	5.61±0.21 5.23±0.24	↑	2.37	0.05	D	plant development, plant growth, epinastic leaves	within plant development models using transgenic *Arabidopsis thaliana* overexpressing *PIN8*: reduced plant growth along with epinastic leaves as developmental defects, the severity of which increases with increasing PIN8 protein content [82]	V
40	BAM1 (821975; AT3G23920)	tmp_3_8641143_G_T:T	atagacgaac	G T	tattagagac	3.67±0.24 4.62±0.28	↓	5.14	10^{-6}	A	food allergenicity	according to an experimental searching for food allergens from Chinese cultivars of the common wheat *Triticum aestivum* L. (1753) by means of the human immunoglobulin E binding capacity using enzyme-linked immunosorbent assay: beta-amylases turned out to	Λ
		tmp_3_8641145_A_T:T	agacgaacgt	A T	ttagagactc	3.67±0.24 4.96±0.26		7.21	10^{-6}	A			

		ENSVATH00344250:T	ataatagacg	A<br>T	acgtattaga	3.67±0.24<br>2.73±0.16	↑	6.79	10^{-6}	A		be among the most powerful food allergens, whose allergenicity for humans increases with the level of expression and *vise versa* [83]	∨

Table S1. *Cont.*

#	*A. thaliana* gene (Entrez ID [37]; TAIR ID [38])	Candidate SNP marker				K_D, nM, MEAN ± SEM, *in silico*					Hand-curated annotation using the PubMed database [39]		
		TAIR SNP ID [38]): minor allele	5′ flank, 10 bp	WT<br>min	3 flank, 10 bp	WT<br>min	Δ	Z	p	ϱ	economically valuable traits	Effect on this trait (ϒ: "∧" as improved; "∨" as impaired) within plant breeding models using *Arabidopsis thaliana* [Ref]	ϒ
41	BAM2 (827959; AT4G00490)	tmp_4_222257_A_C:C	acccttttgt	A<br>C	ctcaacggat	4.09±0.26<br>6.44±0.34	↓	10.82	10^{-6}	A	food allergenicity	according to an experimental searching for food allergens from Chinese cultivars of the common wheat *Triticum aestivum* L. (1753) by means of the human immunoglobulin E binding capacity using enzyme-linked immunosorbent assay: beta-amylases turned out to be among the most powerful food allergens, whose allergenicity for humans increases with the level of expression and *vise versa* [83]	∧
42	BAM5 (827185; AT4G15210)	ENSVATH06672707:C	ttctatataa	A<br>C	gagctcgtga	1.34±0.10<br>1.50±0.11	↓	2.18	0.05	D			∧
		ENSVATH06672712:T	aagtcattat	A<br>T	caatcctcaa	4.11±0.45<br>5.38±0.29		4.47	10^{-3}	B			
		ENSVATH11769885:A	aaagtcatta	T<br>A	acaatcctca	4.11±0.45<br>5.38±0.29		4.47	10^{-3}	B			
		ENSVATH06672714:C	agggcaataa	A<br>C	gtcattatac	4.11±0.45<br>3.61±0.24	↑	2.03	0.05	D			∨
		ENSVATH11769886:T	caataaagtc	A<br>T	ttatacaatc	4.11±0.45<br>3.10±0.21		4.38	10^{-6}	A			
43	BAM6 (817789; AT2G32290)	ENSVATH00256756:A	tgatagcata	C<br>A	gaacgcgcca	6.85±0.41<br>4.50±0.26	↑	10.15	10^{-6}	A			∨
		ENSVATH00256756:T	tgatagcata	C<br>T	gaacgcgcca	6.85±0.41<br>4.28±0.26		11.13	10^{-6}	A			
		ENSVATH07907378:T	aacaaaaata	C<br>T	aaaaaaaaaa	6.85±0.41<br>6.09±0.31		3.01	10^{-2}	C			
		tmp_2_13717083_T_A:T	caaaaataca	A<br>T	aaaaaaaaaa	6.85±0.41<br>3.70±0.21		14.89	10^{-6}	A			
44	BAM7 (819196; AT2G45880)	ENSVATH13650292:C	cattttaata	A<br>C	taataatctc	2.64±0.17<br>1.98±0.13	↑	6.36	10^{-6}	A			∨
45	BMY2 (834566; AT5G45300)	ENSVATH03360456:G	aacgtaattt	A<br>G	taataattac	2.39±0.14<br>4.05±0.25	↓	12.18	10^{-6}	A			∧
		ENSVATH03360457:G	cgtaatttat	A<br>G	ataattaccg	2.39±0.14<br>3.80±0.20		11.65	10^{-6}	A			
		tmp_5_18353506_C_T:T	ttaatattga	C<br>T	gattttgata	4.65±0.30<br>4.21±0.26	↑	2.27	0.05	D			∨
46	BMY3 (831985; AT5G18670)	ENSVATH06989997:C	tttcatctta	T<br>C	ataaacaga	1.70±0.14<br>3.28±0.22	↓	12.15	10^{-6}	A			∧
47	CT-BMY	ENSVATH14232542:A	tgaggtgtaa	G	aaccgtgtga	5.58±0.22	↑	10.20	10^{-6}	A			∨

	(827419; AT4G17090)			A		3.85±0.24							

Table S1. *Cont.*

#	*A. thaliana* gene (Entrez ID [37]; TAIR ID [38])	Candidate SNP marker: TAIR SNP ID [38]): minor allele	5′ flank, 10 bp	WT min	3 flank, 10 bp	K$_D$, nM, MEAN ± SEM, *in silico*: WT min	Δ	Z	p	ϱ	Hand-curated annotation using the PubMed database [39]: economically valuable traits	Effect on this trait (ϒ: "∧" as improved; "∨" as impaired) within plant breeding models using *Arabidopsis thaliana* [Ref]	ϒ
48	A7 (828956; AT4G28395)	ENSVATH14305782:G	aataacctta	A<br>G	tatgtgtagg	3.77±0.23<br>2.88±0.21	↑	5.70	10^{-6}	A	food allergenicity	according to an experimental searching for food allergens from Chinese cultivars of the common wheat *Triticum aestivum* L. (1753) by means of the human immunoglobulin E binding capacity using enzyme-linked immunosorbent assay: albumins turned out to be among the most powerful food allergens, whose allergenicity for humans increases with the level of expression and *vise versa* [83]	∨
49	AT1G05450 (838763)	ENSVATH04524326:G	ttagatatct	A<br>G	tatatcgaca	1.96±0.16<br>3.65±0.24	↓	12.01	10^{-6}	A			∧
		ENSVATH07504033:T	tgtttttttaa	A<br>T	atataatttg	1.96±0.16<br>1.69±0.13	↑	2.63	10^{-2}	C			∨
50	AT1G07747 (5007672)	ENSVATH01022636:T	attttctaag	C<br>T	aagactagaa	5.67±0.34<br>4.28±0.27	↑	6.39	10^{-6}	A			∨
		ENSVATH04536165:C	agcaagacta	G<br>C	aaattagata	5.67±0.34<br>4.81±0.30		3.81	10^{-3}	B			
		tmp_1_2403515_A_T:T	gcaagactag	A<br>T	aattagatag	5.67±0.34<br>3.91±0.24		8.68	10^{-6}	A			
		tmp_1_2403545_C_T:T	cttaaggtta	C<br>T	aacttacaag	5.67±0.34<br>5.06±0.26		2.90	10^{-2}	C			
51	AT1G18280 (838408)	tmp_1_6294116_T_C:C	acactttcta	T<br>C	atgaacaaac	2.84±0.19<br>4.13±0.30	↓	7.75	10^{-6}	A			∧
52	AT1G32280 (840120)	tmp_1_11646138_A_G:G	attactatat	A<br>G	taagatatct	1.18±0.08<br>1.75±0.13	↓	7.71	10^{-6}	A			∧
53	AT1G36150 (840520)	ENSVATH01212636:T	aaaacctaat	A<br>T	gaaaaacaag	4.45±0.27<br>5.91±0.33	↓	6.77	10^{-6}	A			∧
54	AT1G43665 (840951)	ENSVATH04847100:A	cgaagaacta	G<br>A	agtttgattg	7.91±0.36<br>7.04±0.49	↑	2.82	10^{-2}	C			∨
		tmp_1_16453819_G_A:T	gtggcagctg	C<br>T	taaagttata	7.91±0.36<br>4.57±0.26		20.59	10^{-6}	A			
55	AT1G43667 (840952)	tmp_1_16465581_G_A:T	aacattactc	C<br>T	atctatatac	3.52±0.24<br>3.11±0.22	↑	2.54	0.05	D			∨
		tmp_1_16465604_A_T:A	tacctatatt	T<br>A	ggcttattgc	3.52±0.24<br>2.96±0.22		3.40	10^{-3}	B			
56	AT1G62510 (842548)	ENSVATH01439009:T	aggacattca	A<br>T	tatcaaataa	3.05±0.20<br>3.52±0.20	↓	3.31	10^{-3}	B			∧
		ENSVATH01439010:C	caggacattc	A<br>C	tatatcaaat	3.05±0.20<br>3.52±0.20		3.31	10^{-3}	B			
		tmp_1_23137327_T_G:C	acattcatat	A<br>C	tcaaataacc	3.05±0.20<br>3.52±0.20		3.31	10^{-3}	B			

Table S1. *Cont.*

#	*A. thaliana* gene (Entrez ID [37]; TAIR ID [38])	Candidate SNP marker: TAIR SNP ID [38]): minor allele	5′ flank, 10 bp	WT min	3 flank, 10 bp	K_D, nM, MEAN ± SEM, *in silico*: WT min	Δ	Z	p	ϱ	Hand-curated annotation using the PubMed database [39]: economically valuable traits	Effect on this trait (ϒ: "∧" as improved; "∨" as impaired) within plant breeding models using *Arabidopsis thaliana* [Ref]	ϒ
		tmp_1_23137326_A_G:C	cattcatata	T<br>C	caaataacct	3.05±0.20<br>2.75±0.18	↑	2.30	0.05	D			∨
57	AT1G48750 (841297)	ENSVATH04908482:C	atcccctata	A<br>C	atagaaggga	1.85±0.13<br>2.46±0.21	↓	5.22	10^{-6}	A			∧
58	AT4G08670 (826433)	ENSVATH14092599:A	caattcttat	C<br>A	ttttcttttt	4.55±0.25<br>3.53±0.22	↑	6.24	10^{-6}	A			∨
59	AT5G55460 (835639)	tmp_5_22468556_T_A:A	gtccatatca	T<br>A	acttatggtt	3.70±0.24<br>5.92±0.25	↓	12.11	10^{-6}	A			∧
60	AT5G62080 (836328)	ENSVATH07464841:A	tgtaaatctt	G<br>A	tgaactaact	6.77±0.46<br>4.85±0.28	↑	7.41	10^{-6}	A			∨
61	DIR1 (834904; AT5G48485)	ENSVATH14611567:C	ttttccattt	G<br>C	tagtaaaaga	2.24±0.20<br>2.97±0.27	↓	4.33	10^{-3}	B			∧
62	EARLI1 (826860; AT4G12480)	ENSVATH02803678:C	caaatataat	A<br>C	gaacaagttg	2.80±0.19<br>3.29±0.24	↓	3.24	10^{-2}	C			∧
		ENSVATH02803680:G	aatcaaatat	A<br>G	atagaacaag	2.80±0.19<br>3.29±0.24		3.24	10^{-2}	C			
		ENSVATH06633044:T	tgaataatca	A<br>T	atataataga	2.80±0.19<br>1.55±0.11	↑	11.93	10^{-6}	A			∨
		tmp_4_7407065_T_G:C	agggcaaata	A<br>C	ataaataaac	2.80±0.19<br>2.51±0.14		2.54	0.05	D	food allergenicity	according to an experimental searching for food allergens from Chinese cultivars of the common wheat *Triticum aestivum* L. (1753) by means of the human immunoglobulin E binding capacity using enzyme-linked immunosorbent assay: albumins turned out to be among the most powerful food allergens, whose allergenicity for humans increases with the level of expression and *vise versa* [83]	
63	SESA2 (828823; AT4G27150)	ENSVATH06783396:T	ctataaaact	A<br>T	actcttcact	2.38±0.15<br>2.76±0.17	↓	3.43	10^{-3}	B			∧
		tmp_4_13609312_A_C:C	ctcacctata	A<br>C	aactaactct	2.38±0.15<br>3.43±0.23		8.09	10^{-6}	A			
		tmp_4_13609315_C_T:T	acctataaaa	A<br>T	taactcttca	2.38±0.15<br>2.66±0.17		2.53	0.05	D			
64	SESA3 (828824; AT4G27160)	tmp_4_13611767_A_T:T	tcacctataa	A<br>T	tacaaacca	1.69±0.11<br>2.50±0.18	↓	8.14	10^{-6}	A			∧
65	SESA5 (835563; AT5G54740)	ENSVATH03430640:A	tccataaata	T<br>A	attcattccc	2.78±0.24<br>3.82±0.20	↓	6.27	10^{-6}	A			∧
		ENSVATH07420797:C	ataaatatat	T<br>C	cattcccact	2.78±0.24<br>3.79±0.13		6.66	10^{-6}	A			
		tmp_5_22239219_T_C:G	tctcctccat	A<br>G	aatatattca	2.78±0.24<br>3.75±0.28		5.27	10^{-6}	A			
66	XYP2	ENSVATH01769305:T	caataataat	A	tatgaaaaag	1.98±0.14	↓	6.70	10^{-6}	A			∧

#	A. thaliana gene (Entrez ID [37]; TAIR ID [38])	TAIR SNP ID [38]): minor allele	5′ flank, 10 bp	WT<br>min	3 flank, 10 bp	WT<br>min	Δ	Z	p	ϱ	economically valuable traits	Effect on this trait (ϒ: "∧" as improved; "∨" as impaired) within plant breeding models using *Arabidopsis thaliana* [Ref]	ϒ
	(815865; AT2G13820)			T		2.72±0.17							
		tmp_2_5776373_T_A:T	ctttcggcga	A<br>T	aaatataaaa	2.72±0.17<br>2.38±0.14	↑	3.01	10^{-2}	C			∨

Table S1. *Cont.*

#	*A. thaliana* gene (Entrez ID [37]; TAIR ID [38])	Candidate SNP marker: TAIR SNP ID [38]): minor allele	5′ flank, 10 bp	WT<br>min	3 flank, 10 bp	K_D, nM, MEAN ± SEM, *in silico*: WT<br>min	Δ	Z	p	ϱ	Hand-curated annotation using the PubMed database [39]: economically valuable traits	Effect on this trait (ϒ: "∧" as improved; "∨" as impaired) within plant breeding models using *Arabidopsis thaliana* [Ref]	ϒ
67	CRA1 (834435; AT5G44120)	ENSVATH08552658:G	agtcgactct	A<br>G	tatacctgg	1.83±0.14<br>3.52±0.29	↓	11.50	10^{-6}	A	food allergenicity	according to an experimental searching for food allergens from Chinese cultivars of the common wheat *Triticum aestivum* L. (1753) by means of the human immunoglobulin E binding capacity using enzyme-linked immunosorbent assay: globulins turned out to be among the most powerful food allergens, whose allergenicity for humans increases with the level of expression and *vise versa* [83]	∧
		tmp_5_17758377_T_C:G	gactctatat	A<br>G	cctggttctt	1.83±0.14<br>3.53±0.26		12.24	10^{-6}	A			
68	MAG2 (823924; AT3G47700)	ENSVATH02464066:A	acttgatatg	T<br>A	taaaaacatt	3.92±0.28<br>5.01±0.28		5.41	10^{-6}	A			∧
		ENSVATH06262342:C	atttacttga	T<br>C	atgttaaaaa	3.92±0.28<br>5.11±0.24	↓	6.17	10^{-6}	A			
		ENSVATH06262343:G	aatttacttg	A<br>G	tatgttaaaa	3.92±0.28<br>5.11±0.24		6.17	10^{-6}	A			
		ENSVATH06262344:T	aatgccatta	C<br>T	agttttaatt<br>t	3.92±0.28<br>3.53±0.24	↑	2.14	0.05	D			∨
69	AT2G28490 (817397)	tmp_2_12181043_C_T:A	acacatatat	G<br>A	catgcaccct	2.43±0.17<br>1.57±0.11	↑	8.93	10^{-6}	A			∨
70	ZML1 (821670 AT3G21175)	tmp_3_7421965_A_G:G	ttggctaaaa	A<br>G	aggcccaaga	5.91±0.29<br>8.36±0.43	↓	9.73	10^{-6}	A	excessive light stress response, photobleaching, photosystem II photoinactivation	within plant excessive light stress response models using T-DNA-based *ZML1*-knockout *Arabidopsis thaliana* (ecotype Columbia-0): impared photoprotective response to excessive light stress up to photoinactivation of photosystem II and severe photobleaching [84]	∨
		ENSVATH00337517:T	gacgccgttt	G<br>T	gctaaaaaag	5.91±0.29<br>5.25±0.27	↑	3.37	10^{-3}	B	plant fertility, self-incompatibility, stigmatic compatibility	within plant self-incompatibility models using rapeseed (*Brassica napus*) lines overexpressing BnZML1: in self-incompatible stigmas, a partial breakdown of self-incompatibility response as a partial stigmatic compatibility [85]	∧
71	ZML2 (841585; AT1G51600)	ENSVATH01345200:T	tcaacaaaaa	C<br>T	agagaccaat	7.06±0.31<br>7.59±0.31	↓	2.41	0.05	D	excessive light stress response, photobleaching, photosystem II photoinactivation	within plant excessive light stress response models using T-DNA-based *ZML2*-knockout *Arabidopsis thaliana* (ecotype Columbia): impared photoprotective response to excessive light stress up to photoinactivation of photosystem II and severe photobleaching [84]	∨
		ENSVATH04934698:A	agtatttatt	T<br>A	atatctccgg	3.19±0.19<br>2.93±0.18	↑	1.99	0.05	D	plant wound repair, mechanical damage, lignification	within plant wound repair models using maize (*Zea mays*) protoplasts transformed with a vector carrying an additional ZmZML2 gene-copy: ZmZML2 up-regulation can reduce lignification as a plant response to mechanical damage [86]	∨
71	ZPR3 (824443,	ENSVATH04513629:A	ttatttaatt	G<br>T	tattaagatg	2.78±0.18<br>2.49±0.14	↓	2.55	10^{-6}	A	plant development,	within plant development models using transgenic *Arabidopsis thaliana* (ecotype Columbia) overexpressing ZPR3: only newly	∨

AT3G52770)	curled downward leaves	emerging leaves were curled downward after 22 days after stratification [87]

Table S1. *Cont.*

#	*A. thaliana* gene (Entrez ID [37]; TAIR ID [38])	Candidate SNP marker				K_D, nM, MEAN ± SEM, *in silico*					Hand-curated annotation using the PubMed database [39]		
		TAIR SNP ID [38]): minor allele	5′ flank, 10 bp	WT<br>min	3 flank, 10 bp	WT<br>min	Δ	Z	p	ϱ	economically valuable traits	Effect on this trait (ϒ: "∧" as improved; "∨" as impaired) within plant breeding models using *Arabidopsis thaliana* [Ref]	ϒ
72	ZTL (835842, AT5G57360)	ENSVATH03444105:T	tcaacttttt	C<br>T	aataagttgg	4.39±0.24<br>3.13±0.16	↑	9.07	10^{-6}	A	circadian period, hypocotyl length, flowering time	within plant development models using *Arabidopsis thaliana* transfected with plasmid carrying one more ZTL gene copy: ZTL overexpression may be a molecular marker for prolonged circadian period, increased length of hypocotyl, delayed flowering time [88]	∨
		ENSVATH07439752:A	agattttaat	G<br>A	acatttttcc	4.39±0.24<br>3.39±0.20		6.48	10^{-6}	A			
73	ZW18 (842204 AT1G58350)	ENSVATH13630240:G	ccgtgaatat	A<br>G	ttttagcatc	3.71±0.24<br>4.30±0.27	↓	3.28	10^{-2}	C	plant susceptibility to parasites, parasite reprogrammin g of host-plant	within models of plant susceptibility to parasites using transgenic *Arabidopsis thaliana* (ecotype Columbia) susceptable to nematode: both deficiency and excess of certain ZW18 splice variants may be a molecular marker of the nematode reprogramming the normal gene expression pattern of a healthy host-plant for susceptibility to infection by this parasite [89]	∨
		tmp_1_21667503_A_C:G	tcaaatcttt	T<br>G	accgtacgaa	3.71±0.24<br>7.73±0.50		7.33	10^{-6}	A			
		ENSVATH01393400:T	tatattttag	C<br>T	atcaaatga	3.71±0.24<br>2.91±0.22	↑	4.81	10^{-3}	B			
		ENSVATH04993568:A	aagatgccgt	G<br>A	aatatatttt	3.71±0.24<br>2.96±0.20		4.79	10^{-3}	B			
		tmp_1_21667676_G_T:A	gaagatatat	C<br>A	gacttgataa	3.71±0.24<br>2.27±0.23		8.31	10^{-6}	A			
		tmp_1_21667691_G_T:A	attttagcat	C<br>A	aaatgaagat	3.71±0.24<br>3.29±0.19		2.79	10^{-2}	C			
74	ZYP1a (838831, AT1G22260)	tmp_1_7865301_A_G:C	ttctccggta	T<br>C	aaaagaccac	2.33±0.16<br>4.46±0.29	↓	14.00	10^{-6}	A	plant recombination, meiosis, fertility	within plant recombination models using T-DNA-based ZYP1a-knockout *Arabidopsis thaliana* (ecotype Columbia): meiotic defects reducing fertility [90]	∨
		ENSVATH01087323:T	tgctagggtt	G<br>T	cgtttatgaa	5.22±0.27<br>4.73±0.26	↑	2.59	10^{-2}	C		as a heuristic contrast to articles only on various loss-of-function variants of the plant *ZYP1*a and *ZYP1b* genes that we could find in the current state of the PubMed database, which report meiotic defects reducing fertility in such plants [90]: excess ZYP1a might hypothetically contribute to the maintenance of normal meiosis and fertility in plants	∧
		ENSVATH11780374:A	ttgttcttcc	G<br>A	aatagttagc	5.22±0.27<br>4.64±0.33		2.64	10^{-2}	C			
75	ZYP1b (838834, AT1G22275)	ENSVATH11780638:G	cggtataaat	A<br>G	actttatttc	2.21±0.14<br>2.55±0.17	↓	2.98	10^{-2}	C	plant recombination, meiosis, fertility	within plant recombination models using T-DNA-based ZYP1b-knockout *Arabidopsis thaliana* (ecotype Columbia): meiotic defects reducing fertility [90]	∨
		tmp_1_7867104_A_G:G	cttcatcggt	A<br>G	taaataactt	2.21±0.14<br>4.35±0.27		15.11	10^{-6}	A			
		ENSVATH04622206:C	cagattgaaa	T<br>C	gtagctcgtt	8.52±0.32<br>6.38±0.41		7.81	10^{-6}	A		as a heuristic contrast to articles only on various loss-of-function variants of the plant *ZYP1*a and *ZYP1b* genes that we could find in the current state of the PubMed database, which report meiotic defects reducing fertility in such plants [90]: excess ZYP1a might hypothetically contribute to the maintenance of normal meiosis and fertility in plants	∧
		ENSVATH04622207:A	agattgaaat	G<br>A	tagctcgttg	8.52±0.32<br>4.64±0.36	↑	14.04	10^{-6}	A			
		ENSVATH11780637:A	gcgatattct	G<br>A	agtctctgac	9.29±0.60<br>6.55±0.34		8.42	10^{-6}	A			

		tmp_1_7867030_G_A:A	cgttgatcat	G<br>A	ccaagtggcc	9.29±0.60<br>8.20±0.52		2.76	10^{-2}	C			

Table S1. *Cont.*

#	*A. thaliana* gene (Entrez ID [37]; TAIR ID [38])	Candidate SNP marker: TAIR SNP ID [38]): minor allele	5′ flank, 10 bp	WT<br>min	3 flank, 10 bp	K_D, nM, MEAN ± SEM, *in silico*: WT<br>min	Δ	Z	p	ϱ	Hand-curated annotation using the PubMed database [39]: economically valuable traits	Effect on this trait (ϒ: "∧" as improved; "∨" as impaired) within plant breeding models using *Arabidopsis thaliana* [Ref]	ϒ
77	ZFN1 (821230 AT3G02830)	ENSVATH10461609:A	catattcgtc	T<br>A	acgtacatac	3.33±0.26<br>3.94±0.21	↓	3.56	10^{-3}	B	fruit ripening, sucrose content	within plant fruit ripening models using the strawberry (*Fragaria x ananassa*) mutants: FaZFN1 deficit along with reduced sucrose content [91]	∨
		tmp_3_613745_T_G:G	ttcgtctacg	T<br>G	acatacacaa	3.33±0.26<br>5.30±0.23		10.48	10^{-6}	A			
78	ZFN2 (817855 AT2G32930)	ENSVATH07908096:C	aatttattaa	T<br>C	ccgtacacgg	3.88±0.28<br>4.39±0.31	↓	2.45	0.05	D	fruit ripening, sucrose content	within plant fruit ripening models using the strawberry (*Fragaria x ananassa*) mutants: FaZFN2 deficit along with reduced sucrose content [91]	∨
79	ZFN3 (831516, AT5G16540)	ENSVATH06976768:G	catacatact	A<br>G	accaaatcag	2.71±0.21<br>3.21±0.24	↓	3.19	10^{-2}	C	flower development, flowering time	according to the only article that we could find in the current state of the PubMed database, which has empirically concluded based on the combined analysis of gene expression data of *Arabidopsis thaliana* development in comparison with plant gene phylogeny in both *Arabidopsis thaliana* and *Oryza sativa*: ZFN3 excess and deficit might positive and negative effect, respectively, on both flower development and on flowering time [92]	∨
		ENSVATH06976772:C	tagttttttt	T<br>C	ctttcttttg	7.19±0.33<br>6.68±0.33	↑	2.17	0.05	D			∧
80	ZFP10 (818351, AT2G37740)	ENSVATH13564244:C	tttaatttc	T<br>C	ttttatataa	1.88±0.12<br>2.10±0.13	↓	2.39	0.05	D	plant growth, dwarfing, leafs development, flowering time	as a heuristic contrast to the only article that we could find in the current state of the PubMed database, which reports about plant development models using transgenic both Arabidopsis thaliana and Tobacco, which overexpress ZFP10 along with dwarfing, abnormal leafs and flowering as early as higher ZFP10 abundance: ZFP10 deficit might hypothetically contribute to the maintenance of normal plant growth, leafs development and flowering time [93]	∧
81	ZFP11 (818842, AT2G42410)	ENSVATH01980798:A	tttatttttt	T<br>A	aaataatttc	3.58±0.17<br>4.21±0.19		4.90	10^{-6}	A	stem elongation, leaf shape, fertility, sterility	as a heuristic contrast to the only article that we could find in the current state of the PubMed database, which reports about plant development models using transgenic both *Arabidopsis thaliana* and Tobacco, which overexpress ZFP11 along with reduced stem elongation, abnormal leaf shape, and sterility: ZFP11 deficit might hypothetically contribute to the maintenance of normal stem elongation, leaf shape, and fertility [94]	∧
		ENSVATH05703253:C	ttttttttaaa	T<br>C	aatttccact	3.58±0.17<br>4.28±0.22	↓	5.06	10^{-6}	A			
		ENSVATH13618097:C	attttatttt	T<br>C	ttaaataatt	3.58±0.17<br>3.85±0.21		2.02	0.05	D			
		ENSVATH05703252:T	ttattttttt	A<br>T	aataatttcc	3.58±0.17<br>3.31±0.18		2.16	0.05	D		within plant development models using transgenic both *Arabidopsis thaliana* and Tobacco, which overexpress ZFP11: reduced stem elongation, abnormal leaf shape, and sterility [94]	∨
		ENSVATH14608579:T	tattttttta	A<br>T	ataatttcca	3.58±0.17<br>1.89±0.11		16.84	10^{-6}	A			
		tmp_2_17657744_A_ATT:T	tttttatttt	A<br>T	ttttttttaaa	3.58±0.17<br>3.32±0.17		2.15	0.95	D			
82	ZFP2 (835856,	ENSVATH07440609:T	tagaaaaagg	C<br>T	atacattaac	4.47±0.15<br>3.96±0.24	↑	3.52	10^{-3}	B	flower abscission,	within plant development models using transgenic *Arabidopsis thaliana*, which overexpress *ZFP2*: asynchronous and delayed abscission of flower	∨

	AT5G57520)										flower morphology, pollination, fertility	parts with changes in morphology that reduced both pollination and fertility [95]	

Table S1. *Cont.*

#	*A. thaliana* gene (Entrez ID [37]; TAIR ID [38])	Candidate SNP marker: TAIR SNP ID [38]): minor allele	5′ flank, 10 bp	WT min	3 flank, 10 bp	K_D, nM, MEAN ± SEM, *in silico*: WT min	Δ	Z	p	ϱ	Hand-curated annotation using the PubMed database [39]: economically valuable traits	Effect on this trait (ϒ: "Λ" as improved; "V" as impaired) within plant breeding models using *Arabidopsis thaliana* [Ref]	ϒ
83	ZFP3 (832587, AT5G25160)	ENSVATH07038344:A	cacacttaat	G A	tatttgttgg	4.99±0.26 2.62±0.15	↑	16.31	10^{-6}	A	semidwarf growth habit, fertility, light sensitivity, hypocotyl elongation	within plant development models using transgenic *Arabidopsis thaliana* overexpressing *ZFP3*: semidwarf growth habit, defects in fertility, and enhanced sensitivity of hypocotyl elongation to red but not to far-red or blue light [96]	V
		ENSVATH07038345:A	ttaatgtatt	T A	gttggactat	4.99±0.26 3.38±0.22		9.34	10^{-6}	A			
		ENSVATH11025805:G	tacacactta	A G	tgtatttgtt	4.99±0.26 4.36±0.25		3.45	10^{-3}	B			
		tmp_5_8687115_C_A:A	ccaaataaat	C A	tgaaccggaa	4.99±0.26 4.08±0.18		5.80	10^{-6}	A			
		tmp_5_8687157_T_A:A	acacacttaa	T A	gtatttgttg	4.99±0.26 3.64±0.21		8.09	10^{-6}	A			
84	ZFP4 (842928, AT1G66140)	ENSVATH01479027:A	tacatgatta	T A	atgtattttt	2.65±0.17 3.89±0.19	↓	9.32	10^{-6}	A	hypersensitivity to abscisic acid, germination	within plant development models using T-DNA-based double *ZFP3-ZFP4* knockout *Arabidopsis thaliana* treated with abscisic acid: hypersensitivity to abscisic acid reducing germination efficiency [96]	V
		tmp_1_24622162_A_T:A	cttacatgat	T A	atatgtattt	2.65±0.17 3.89±0.19		9.38	10^{-6}	A			
		ENSVATH01479026:A	atgattatat	G A	tattttttatt	2.65±0.17 1.64±0.13	↑	9.21	10^{-6}	A	hypocotyl lengths, seedlings development	within plant development models using transgenic *Arabidopsis thaliana* overexpressing *ZFP4*: reduced hypocotyl lengths of germinated seedlings [96]	V
85	ZFP5 (837588, AT1G10480)	tmp_1_3449540_A_T:T	catgactaat	A T	taaaaaaccc	1.84±0.12 2.59±0.17	↓	7.32	10^{-6}	A	trichome number	within plant development models using ZFP5 loss-of-function mutant *Arabidopsis thaliana*: a reduced number of trichomes on sepals, cauline leaves, paraclades, and main inflorescence stems [97]	V
		tmp_1_3449548_C_AT:T	atataaaaaa	C T	ccttcataca	1.84±0.12 2.07±0.13		2.54	0.05	D			
86	ZFP7 (839077, AT1G24625)	tmp_1_8727258_T_C:G	agacactttt	A G	ttctcccatg	4.39±0.23 5.68±0.31	↓	6.74	10^{-6}	A	hypocotyl lengths, seedlings development	as a heuristic contrast to the only article that we could find in the current state of the PubMed database, which reports about plant development models using transgenic Arabidopsis thaliana overexpressing ZFP7 along with reduced hypocotyl lengths of germinated seedlings: ZFP7 deficit might hypothetically contribute to the maintenance of normal hypocotyl lengths of germinated seedlings [96]	Λ
		tmp_1_8727262_A_G:C	tggcagacac	T C	tttattctcc	4.39±0.23 5.68±0.31		6.74	10^{-6}	A			
87	ZFP8 (818794, AT2G41940)	ENSVATH05701009:G	tctaatatac	T G	aatgaaaaaa	3.65±0.24 4.09±0.19	↓	2.83	10^{-2}	C	trichome density	within plant development models using *ZFP8* loss-of-function mutant *Arabidopsis thaliana*: reduced trichome density on upper cauline leaves and branches [98]	V
88	ZIF1 (831219, AT5G13740)	ENSVATH03054081:T	tcttaaatac	C T	agttttttaat	3.65±0.16 2.83±0.18	↑	6.65	10^{-6}	A	Zn tolerance, Zn retention in roots, interveinal leaf chlorosis	within plant development models using *Arabidopsis thaliana* overexpressing *ZIF1*: increased Zn tolerance leading Zn retention in roots along with interveinal leaf chlorosis [99]	V
		ENSVATH03054083:G	cagtttttaa	T G	tacaaattac	3.65±0.16 3.20±0.20		3.44	10^{-3}	B			
		ENSVATH06963789:T	atcttttttta	A T	aaaaaaatatg	3.79±0.19 2.41±0.16		10.85	10^{-6}	A			

		tmp_5_4432222_C_A:A	tcggactata	C<br>A	aattaatatc	3.65±0.16<br>2.81±0.18		6.77	10^{-6}	A
		tmp_5_4432295_A_T:T	tcttttttaa	A<br>T	aaaaatatgt	3.79±0.19<br>2.86±0.16		7.50	10^{-6}	A

Table S1. *Cont.*

#	*A. thaliana* gene (Entrez ID [37]; TAIR ID [38])	Candidate SNP marker				K_D, nM, MEAN ± SEM, *in silico*					Hand-curated annotation using the PubMed database [39]		
		TAIR SNP ID [38]): minor allele	5′ flank, 10 bp	WT<br>min	3 flank, 10 bp	WT<br>min	Δ	Z	p	ϱ	economically valuable traits	Effect on this trait (ϒ: "∧" as improved; "∨" as impaired) within plant breeding models using *Arabidopsis thaliana* [Ref]	ϒ
89	ZIFL1 (831220, AT5G13750)	ENSVATH14020110:C	atcatcatca	T<br>C	attataaaaa	1.87±0.12<br>2.19±0.14	↓	3.46	10^{-3}	B	root length, cobalt sensitivity	within plant development models using *Arabidopsis thaliana* carrying disrupted *ZIFL1*gene: reduced root length and decreased sensitivity to inhibitory cobalt [100]	∨
		tmp_5_4437787_T_G:G	catcatatta	T<br>G	aaaaatggtg	1.87±0.12<br>3.96±0.35		14.00	10^{-6}	A			
90	ZIFL2 (823490, AT3G43790)	ENSVATH02391036:T	aataattatc	C<br>T	taaaaacaa	6.10±0.38<br>5.03±0.26	↑	4.76	10^{-3}	B	resistance to cation cesium toxicity, potassium responsiveness, plant growth, plant development	within plant development models using T-DNA-based *ZIFL1*-knockout *Arabidopsis thaliana*: reduced sensitivity to cation cesium as toxic for plant, as well as improved responsiveness to potassium as an essential mineral nutrient for plant growth and development [101]	∧
		ENSVATH06184766:T	caacaccact	G<br>T	agatatttta	5.00±0.30<br>4.25±0.24		3.95	10^{-3}	B			
		ENSVATH06184768:T	tatttggcgc	C<br>T	tttagaataa	6.10±0.38<br>5.22±0.31		3.64	10^{-3}	B			
		ENSVATH14318785:T	aattttgttt	G<br>T	taaaacaaca	5.00±0.30<br>4.53±0.22		2.56	0.05	D			
		ENSVATH14318786:A	gatattttat	T<br>A	tggttctctt	5.00±0.30<br>2.88±0.19		12.47	10^{-6}	A			
		tmp_3_15655250_T_G:G	aaaattttgt	T<br>G	tgtaaaacaa	5.00±0.30<br>4.49±0.29		2.48	0.05	D			
		tmp_3_15655356_A_T:T	tccgaagaaa	A<br>T	taattatcct	6.10±0.38<br>5.63±0.29		1.98	0.05	D			
91	ZIGA4 (837390, AT1G08680)	ENSVATH01025652:A	agaaaaggag	T<br>A	attaatggaa	2.58±0.19<br>4.52±0.30	↓	11.32	10^{-6}	A	drought stress response	within plant stress-response models using rice (*Oryza sativa* L.) under drought stress in comparison with a norm by means of whole-genome transcriotome profiling (i.e., RNA-Seq data): ZIGA4 downregulation may be a molecular marker for plant response to drought stress [102]	∧
		tmp_1_2762564_G_C:C	gagtattaat	G<br>C	gaaaacatgt	2.58±0.19<br>3.03±0.22		3.08	10^{-2}	C			
92	ZIP1 (820457, AT3G12750)	tmp_3_4053228_C_TCT:A	tctataaaaa	G<br>A	ctcctctttc	2.12±0.14<br>2.32±0.15	↓	2.02	0.05	D	plant stress response, Zn-toxicity stress	within plant stress response models using a mutant line of Arabidopsis thaliana with a vacuolar membrane defect sensitive to zinc: the expression level of ZIP1 was markedly reduced, to 30% of the control, as plant response to Zn-toxicity stress [103]	∧
		tmp_3_4053233_T_A:T	ttctctctat	A<br>T	aaaagctcct	2.12±0.14<br>2.75±0.18		5.76	10^{-6}	A			
93	ZIP10 (840014, AT1G31260)	ENSVATH14930063:C	ttgtcttata	T<br>C	aagaagatta	1.69±0.13<br>2.74±0.18	↓	9.41	10^{-6}	A	plant stress response, high-salt stress	within plant stress response models using mature *Arabidopsis thaliana* (ecotype Columbia) shoot tissue from salt-treated plants: *ZIP10* downregulation is a molecular marker for plant response to high-salt stress [104]	∧
94	ZIP11 (842041, AT1G55910)	ENSVATH04513629:A	ttatttaatt	G<br>T	tattaagatg	2.78±0.18<br>2.49±0.14	↓	2.55	10^{-6}	A	plant-environment response, environmental iron (Fe) deficit	within plant-environment response models using rice (*Oryza sativa*): *ZIP11* upregulation may be a molecular marker for plant response to environmental iron (Fe) deficit rather than those for zinc (Zn), copper (Cu) and manganese (Mn) [105]	∧
95	ZIP2 (836071	ENSVATH03453066:C	tatgtacttt	T<br>C	tgtagtacaa	3.33±0.21<br>4.38±0.26	↓	6.27	10^{-6}	A	plant development, severe leaf chlorosis,	within plant development models using Arabidopsis thaliana defective in Zn/Fe-transport system under Zn/Fe deficit stess : ZIP2	∨

	AT5G59520)										lethality in soil, defective leaves, defective stems, defective roots, Zn/Fe deficit stress	downregulation in roots along with severe leaf chlorosis and lethality in soil as well as reduced chloroplast thylakoid stacking into grana, lack of palisade parenchyma differentiation in leaves, reduced number of vascular bundles in stems, irregular patterns of enlarged endodermal and cortex cells in roots [106]	

Table S1. *Cont.*

#	*A. thaliana* gene (Entrez ID [37]; TAIR ID [38])	Candidate SNP marker				K$_D$, nM, MEAN ± SEM, *in silico*					Hand-curated annotation using the PubMed database [39]		
		TAIR SNP ID [38]): minor allele	5′ flank, 10 bp	WT min	3 flank, 10 bp	WT min	Δ	Z	p	ϱ	economically valuable traits	Effect on this trait (ϒ: "Λ" as improved; "V" as impaired) within plant breeding models using *Arabidopsis thaliana* [Ref]	ϒ
96	ZIP4 (842113, AT1G56590)	tmp_1_21204893_A_T:A	ttaaaaacta	T A	agaaggccca	3.31±0.23 4.12±0.17	↓	5.45	10^{-6}	A	plant development, amyloplasts, vacuolar membrane, endodermis, inflorescence stems	within plant development models using transgenic ZIP4 loss-of-function Arabidopsis thaliana: abnormal amyloplasts and defective vacuolar membrane in living endodermal cells of inflorescence stems [107]	V
		tmp_1_21204897_T_C:G	aacgttaaaa	A G	ctatagaag	3.31±0.23 4.25±0.30		5.13	10^{-6}	A			
97	ZIP5 (837029 AT1G05300)	ENSVATH01014951:T	agtatataat	A T	taacaaaaaa	1.56±0.10 1.72±0.13		1.98	0.05	D	cadmium (Cd) tolerance, seed mineral concentrations	within plant seed development models using *ZIP5* knockout *Arabidopsis thaliana*: increased tolerance to cadmium (Cd) and decreased seed mineral concentrations [108]	V
		ENSVATH04523437:G	atattaaagt	A G	tataatataa	1.56±0.10 2.59±0.09	↓	13.53	10^{-6}	A			
		ENSVATH10632198:A	catattaaag	T A	atataatat	1.56±0.10 2.60±0.17		11.03	10^{-6}	A			
98	ZIP9 (829439 AT4G33020)	ENSVATH06815813:A	ccttgttaac	G A	tacgtgcacg	5.00±0.23 4.15±0.23	↓	5.09	10^{-6}	A	stress response; iron (Fe) deficiency stress	within plant stress response models using *Arabidopsis thaliana* under iron (Fe) deficient growth conditions: ZIP9 upregulation in both in shoot and roots may be a molecular marker for plant response to iron (Fe) deficiency stress [109]	Λ
99	Z-ISO (837626, AT1G10830)	ENSVATH04553459:G	agattttatt	A G	tagttgtagt	2.22±0.14 2.74±0.17		4.21	10^{-3}	B	fruit ripening, breaker stage, red-ripe stage, lutein excess, yellow pigment, ethylene deficit, fruit ripening regulator	within plant fruit ripening models using proteom profiling of mature green (MG), breaker (BR) and red-ripe (RR) stages of tomato (*Solanum lycopersicum*) mutants: SlZ-ISO downregulation in both breaker and red-ripe stages along with excess of lutein as the main yellow pigment and deficit of ethylene as an important regulator of fruit ripening [110]	V
		ENSVATH04553459:T	agattttatt	A T	tagttgtagt	2.22±0.14 2.74±0.17	↓	4.21	10^{-3}	B			
		tmp_1_3607591_A_T:A	gagattttat	T A	atagttgtag	2.22±0.14 2.54±0.15		3.15	10^{-2}	C			
100	ZKT (841995, AT1G55480)	tmp_1_20713462_G_T:T	cacttgctct	G T	aacgttacca	7.76±0.44 4.73±0.22	↓	13.45	10^{-6}	A	early stress response; high-salt stress, drought stress	within plant stress response models using proteome analyses of rapeseed (*Brassica napus*) seedlings pretreated with 245 mM NaCl or 25% polyethylene glycol to imitate environment salt and drought stresses: BnZKT upregulation may be a molecular marker for early responses of plants to salt and drought [111]	Λ
101	2A6 (838763; AT1G03410)	ENSVATH04513629:A	ttatttaatt	G A	tattaagatg	2.78±0.18 2.49±0.14	↑	2.55	0.05	D	ethylene responsiveness	according to the identification of this gene for the first time *via* searching for homology to the tomato gene E8 in a cDNA library of *A. thaliana*: 2A6 excess may resist ethylene response in etiolated seedlings, leaves, stems and flowers [112]	V
102	2-Cys Prx B	tmp_5_1919025_T_A:A	tatttgtatt	T	tcttaattat	4.39±0.19	↑	7.51	10^{-6}	A	photosynthetic	as an heuristic contrast to plant development models using	Λ

#	A. thaliana gene	TAIR SNP ID: minor allele	5′ flank, 10 bp	WT min	3 flank, 10 bp	WT min	Δ	Z	p	ϱ	economically valuable traits	Effect on this trait	ϒ
	(830517; AT5G06290)			A		3.34±0.19					efficiency	Arabidopsis thaliana 2-Cys Prx B knockout: 2-Cys Prx B excess may increase photosynthetic efficiency under high-light growth conditions [113]	
		ENSVATH03029898:A	ttttcttaat	T<br>A	attatgattg	4.39±0.19<br>3.80±0.23		3.81	10^{-3}	B			
		ENSVATH13940029:C	ttcttaatta	T<br>C	tatgattgga	4.39±0.19<br>3.81±0.28		3.32	10^{-3}	B			

Table S1. *Cont.*

#	*A. thaliana* gene (Entrez ID [37]; TAIR ID [38])	Candidate SNP marker				K_D, nM, MEAN ± SEM, *in silico*					Hand-curated annotation using the PubMed database [39]		
		TAIR SNP ID [38]): minor allele	5′ flank, 10 bp	WT min	3 flank, 10 bp	WT min	Δ	Z	p	ϱ	economically valuable traits	Effect on this trait (ϒ: "∧" as improved; "∨" as impaired) within plant breeding models using *Arabidopsis thaliana* [Ref]	ϒ
103	3BETAHSD/D1 (841132; AT1G47290)	ENSVATH04886215:T	atttataaat	A<br>T	taagtattct	1.96±0.11<br>2.27±0.13	↓	3.63	10^{-3}	B	phytosterol overproduction	within the framework of a comprehensive experimental and bioinformatic study of the reticulon gene family and the proteins encoded by them: 3BETAHSD/D1 heterozygous mutant lines show increased sterol levels in roots and leaves [114]	∧
		ENSVATH01304136:T	aaaattaaac	G<br>T	aataaattat	5.36±0.23<br>4.24±0.19	↑	7.65	10^{-6}	A	narrow endoplasmic reticulum	within the framework of a comprehensive experimental and bioinformatic study of the reticulon gene family and the proteins encoded by them: 3BETAHSD/D1 excess can narrow the endoplasmic reticulum [114]	∨
		ENSVATH01304139:C	gatttcaaaa	A<br>C	ataaaataaa	5.36±0.23<br>4.12±0.22		7.65	10^{-6}	B			
		ENSVATH04886220:T	tttgttggca	G<br>T	aaaattaaac	5.36±0.23<br>4.72±0.19		4.36	10^{-3}	A			
		ENSVATH04886221:T	taaacgaata	A<br>T	attatgcaat	5.36±0.23<br>3.04±0.23		13.04	10^{-6}	A			
		ENSVATH14241813:A	attcagattt	C<br>A	aaaaaataaa	5.36±0.23<br>4.19±0.22		7.38	10^{-6}	A			
		ENSVATH13402286:G	tttataaata	T<br>G	aagtattctt	1.96±0.11<br>1.80±0.12		2.03	0.05	D			
**104**	4CL3 (842814; AT1G65060)	ENSVATH01466772:A	aaaacacgca	C<br>A	atttctatat	1.90±0.13<br>2.18±0.16	↓	2.78	10^{-2}	C	infection resistance	within plant innate immunity models using *Arabidopsis thaliana* 4cl3-deficient mutants *in vivo*: improved resistance against *Pseudomonas syringae* [115]	∧
		ENSVATH13728248:C	cacatttcta	T<br>C	atattattta	1.90±0.13<br>3.09±0.14		11.81	10^{-6}	A			
		tmp_1_24171657_T_GC	ccacacattt	A<br>C	tttcttcact	4.45±0.24<br>7.32±0.53		11.10	10^{-6}	A			
		tmp_1_24171580_G_T:A	acgcacattt	C<br>A	tatatattat	1.90±0.13<br>1.48±0.08	↑	5.71	10^{-6}	A	cold tolerance	within plant cold tolerace models using rapeseed subjected with T-DNA insertion carrying homologous *A. thaliana* 4cl3 gene: improved cold tolerance [116]	∨
105	4CL5 (821677; AT3G21230)	ENSVATH00337714:G	atggcttata	T<br>G	acaaaatcat	1.40±0.10<br>3.98±0.29	↓	19.96	10^{-6}	A	phytomedication	according to a phytopharmaceutical study on how to produce natural phytomedications using super-producing yeasts carrying plant genes: 4cl5 deficiency in plant reduces content of natural avenanthramides with antioxidant, anti-inflammatory, and antiproliferative bioactivities in plant foods [117]	∨

106	4CL8 (833792; AT5G38120)	tmp_5_15213636_T_G:G	agtgaatatt	T<br>G	atagtaaata	1.64±0.11<br>2.89±0.15	↓ 13.21	10^{-6}	A	herbal food supplements, coenzyme Q10	within economically valuable plant breeding models using T-DNA-based 4cl8-knockout *Arabidopsis thaliana*: compared with wild-type plants, 20% decreased content of ubiquinone, which is the herbal food supplement coenzyme Q10 [118]	V
		ENSVATH12149211:T	cactaaaata	C<br>T	aaatattcgt	4.06±0.23<br>2.34±0.15	↑ 12.89	10^{-6}	A		within economically valuable plant breeding models using T-DNA-based 4cl8-knockin Arabidopsis thaliana: compared with wild-type plants, 50% increased content of ubiquinone as the herbal food supplement coenzyme Q10 [118]	Λ
107	5-FCL (831144; AT5G13050)	ENSVATH03052038:A	tgtcgtttat	G<br>A	gtttggaata	5.58±0.29<br>3.23±0.23	↑ 12.38	10^{-6}	A	growth rate, flowering	as a heuristic contrast to the only economically valuable plant trait breeding model using the T-DNA-based 5-fcl knockout *Arabidopsis thaliana* (ecotype Columbia): increased growth rate and accelerated flower onset [119]	Λ

Table S1. *Cont.*

#	*A. thaliana* gene (Entrez ID [37]; TAIR ID [38])	Candidate SNP marker				K_D, nM, MEAN±SEM, *in silico*					Hand-curated annotation using the PubMed database [39]		
		TAIR SNP ID [38]): minor allele	5′ flank, 10 bp	WT<br>min	3 flank, 10 bp	WT<br>min	Δ	Z	p	ϱ	economically valuable traits	Effect on this trait (ϒ: "∧" as improved; "∨" as impaired) within plant breeding models using *Arabidopsis thaliana* [Ref]	ϒ
108	5PTASE11 (841160; AT1G47510)	ENSVATH01308444:T	`tggtttctat`	A<br>T	`tatacaagta`	1.05±0.10<br>1.49±0.11	↓	5.99	10^{-6}	A	seed germination, seedling hypocotyl growth	within economically valuable plant breeding models using T-DNA-related 5ptase11 loss-of-*function Arabidopsis thaliana* mutant line Salk (ecotype Columbia): compared with wild-type plants, slowed seed germination and decreased seedling hypocotyl growth [120]	∨
		ENSVATH04890271:T	`acgttggttt`	C<br>T	`tatatataca`	1.05±0.10<br>1.44±0.09		5.58	10^{-6}	A			
		ENSVATH13411044:G	`atatatatat`	A<br>G	`tatgaatatt`	1.18±0.10<br>1.43±0.10		3.45	10^{-3}	B			
		tmp_1_17438445_T_G:C	`atcaatatat`	A<br>C	`tatatatgaa`	1.18±0.10<br>1.63±0.13		5.50	10^{-6}	A			
		ENSVATH07633212:T	`tatatatatg`	A<br>T	`atattgaaga`	3.75±0.25<br>3.23±0.24	↑	2.97	10^{-2}	C	development of flowers, leaves, roots, siliques, bolts, seedlings	within plant auxin response models using Arabidopsis tissues treated with auxin and, next, studied with qPCR: auxin upregulates 5ptase11 in flowers, rosette leaves, cauline leaves, roots, siliques, bolts, and seedlings along with corresponding improvements in their development [121]	∧
		tmp_1_17438527_G_A:T	`tctttgtagt`	G<br>T	`aacaccattt`	4.54±0.25<br>3.00±0.21		8.84	10^{-6}	A			
109	5PTASE13 (837069; AT1G05630)	ENSVATH10657714:T	`aaacaaaaac`	A<br>T	`ttcgtttttt`	9.87±0.45<br>10.74±0.47	↓	2.70	10^{-2}	C	root gravitropism, seed germination, seedling growth	within economically valuable plant breeding models using 5ptase13-deficient *Arabidopsis thaliana* line 5pt13 (ecotype Columbia): root gravitropism hypersensitivity, extended expression of the auxin efflux carrier PIN2, resistance to inhibitors of both seedling growth and intracellular circulation of PIN proteins [122]	∧
		tmp_1_1682324_A_G:G	`tcgtcttcat`	A<br>G	`tttctctctc`	6.94±0.36<br>9.25±0.68		6.40	10^{-6}	A			
		ENSVATH04525467:A	`tctcaacttt`	G<br>A	`actgcgtctt`	9.87±0.45<br>5.06±0.28	↑	18.70	10^{-6}	A	root growth, low-nutrient conditions	within economically valuable plant breeding models using 5ptase13-deficient *Arabidopsis thaliana* line transfected with vector carrying an aditional normal copy of this gene: improved root growth as an additive complementation to the initializing 5ptase13-deficient plant with decreased root growth at the low-nutrient conditions [123]	∧
		ENSVATH10657775:A	`aacaaaaaca`	T<br>A	`tcgttttttt`	9.87±0.45<br>9.02±0.41		2.79	10^{-2}	C			

Table S2. Significant correlations between *in silico* estimates of the equilibrium dissociation constant K_D of an arbitrary plant TBP-promoter complexes, which were documented within the AtSNP_TATAdb database [this work], on one side and strength values of the same promoters, which were independently experimentally measured *in vivo* [125], on another side that are robust to variations in statistical criteria examined, genomic environments and plant species under study as well as their tissues and growing conditions.

	**AtSNP_TATAdb [this work]**		**Promoter strength (log_2-values; normalized to the 35S minimal promoter), *in vivo* magnitudes [125]**						
#	Transcript, Ensembl Plants ID [31]	K_D, nM, *in silico*	*Gene*, TAIR ID [38]	no enhancer, tobacco leaves, light	with enhancer, tobacco leaves, light	no enhancer, tobacco leaves, dark	with enhancer, tobacco leaves, dark	no enhancer, maize protoplasts, dark	with enhancer, maize protoplasts, dark
1	EMB3101-201	3.35	AT1G05600	-0.69	-1.02	-1.02	-0.31	-3.92	-2.29
2	NGA3-201	2.52	AT1G01030	-2.03	-0.19	-1.35	-0.56	-1.94	-1.08
3	IQD18-201	1.85	AT1G01110	-1.03	1.99	-0.81	2.58	-1.58	0.18
4	AT1G01130-201	6.08	AT1G01130	-2.56	0.52	-1.85	0.98	-2.83	-0.51
5	GIF2-201	2.93	AT1G01160	-1.42	1.22	-1.91	0.86	-3.11	-2.37
6	CYP78A8-202	3.56	AT1G01190	0.62	3.64	-2.61	4.02	-0.42	1.33
7	FKGP-204	5.21	AT1G01220	-2.86	0.01	-1.63	0.92	-3.03	-1.75
8	AT1G01225-201	4.88	AT1G01225	-3.07	0.92	-2.99	1.00	-3.05	-2.12
9	AT1G01230-201	4.18	AT1G01230	-2.28	1.33	-1.70	1.06	-3.36	-2.31
10	AT1G01240-201	6.24	AT1G01240	-2.22	-1.22	-1.15	-0.17	-2.68	-1.90
11	AT1G01260-201	4.89	AT1G01260	-1.79	0.32	-1.14	0.28	-2.80	-1.41
12	AT1G01355-201	5.44	AT1G01355	-1.77	2.39	-1.56	2.42	-2.32	-0.89
13	HTR12-201	2.85	AT1G01370	-2.74	-0.86	-2.35	-1.01	-3.34	-2.28
14	AT1G01440-201	2.17	AT1G01440	-1.25	2.37	-2.36	1.99	-2.27	0.60
15	AT1G01500-201	4.50	AT1G01500	-0.13	1.08	-1.51	1.23	-2.43	-0.75
16	AGL28-201	1.72	AT1G01530	-1.15	3.06	0.32	3.94	-2.83	-0.28
17	AT1G01540-201	1.56	AT1G01540	0.20	3.53	0.01	3.30	-2.32	0.75
18	BPS1-201	3.55	AT1G01550	-0.18	0.44	0.28	0.29	-1.78	-1.00
19	AT1G01640-201	4.00	AT1G01640	-1.77	2.99	-2.07	2.99	-1.83	-0.25
20	SPPL4-201	3.80	AT1G01650	-2.05	1.02	0.81	2.07	-1.85	-0.59
21	AT1G01670-201	2.90	AT1G01670	-1.39	2.50	-1.11	2.61	-1.93	-0.76
22	PRD3-201	1.86	AT1G01690	0.56	1.65	-1.03	1.41	-1.77	-0.27
23	AT1G01730-201	10.49	AT1G01730	-2.76	-0.35	-2.74	-0.17	-3.49	-1.27
24	AT1G01760-202	9.61	AT1G01760	-2.25	-1.06	-2.31	-0.64	-1.43	-0.10
25	AT1G01770-201	1.08	AT1G01770	-2.64	1.56	-1.67	1.26	-2.67	-1.48
26	PLIM2b-201	2.12	AT1G01780	0.05	1.51	-0.18	1.40	-1.80	0.42
27	PEX11C-201	4.45	AT1G01820	-1.54	1.78	-1.79	2.27	-3.17	-1.94
28	AT1G01830-203	1.77	AT1G01830	-2.34	0.86	-2.22	1.68	-2.71	-0.54
29	EDA10-201	7.07	AT1G01960	-2.14	1.20	-1.99	0.65	-3.47	-2.88
30	GAE2-201	2.37	AT1G02000	-2.51	0.93	-2.49	0.89	-3.45	-2.38
31	VAD1-201	7.14	AT1G02120	-2.56	-2.33	-3.34	-1.05	-7.29	-5.50
32	AT1G02150-201	5.53	AT1G02150	-2.32	1.10	-2.33	1.76	-3.82	-0.72
33	AT1G02160-201	1.71	AT1G02160	-0.71	1.49	-0.42	2.62	-2.34	-0.32
34	NAC004-201	2.10	AT1G02230	2.77	2.43	2.17	2.92	-2.42	-0.24
35	TOC33-201	3.03	AT1G02280	-0.23	0.92	-0.69	1.62	-3.41	-1.63
36	MAN1-202	4.03	AT1G02310	-1.94	1.14	-1.55	1.46	-2.93	-1.89
37	AT2G01060-201	6.94	AT2G01060	-2.69	-0.37	-3.47	-0.20	-3.12	-1.15
38	AT2G14700-201	2.12	AT2G14700	-2.51	1.73	-1.17	2.28	-2.18	0.50
39	AT2G20250-201	3.41	AT2G20250	3.99	3.98	1.53	3.91	-0.76	0.99
40	AT2G40004-201	1.19	AT2G40004	-1.46	2.60	-1.30	2.90	-1.23	0.29
41	RGF4-201	3.21	AT3G30350	-1.82	1.37	-1.19	1.83	-3.83	-2.69
42	AT3G42850-201	4.45	AT3G42850	-1.07	0.69	-1.72	1.21	-2.65	-1.10
43	AT3G49950-201	3.96	AT3G49950	-1.58	-0.48	-2.69	0.65	-2.36	-1.62
44	SAP130b-201	2.95	AT3G55220	-1.75	1.07	-2.38	0.61	-4.30	-2.02
45	AT4G10110-201	4.77	AT4G10110	-2.87	-0.57	-4.81	-0.15	-4.11	-3.30
46	AT4G22110-202	2.25	AT4G22110	-0.18	1.70	-0.85	1.79	-2.44	-0.88
47	AT4G40000-201	3.88	AT4G40000	-1.17	1.81	-0.51	1.94	-2.67	-0.64
48	AT5G11700-201	5.08	AT5G11700	-2.56	-1.11	-2.59	-0.97	-3.20	-1.67
49	GolS10-201	5.96	AT5G30500	-0.78	1.93	0.36	2.65	-0.86	1.47
50	AT5G52800-201	9.20	AT5G52800	-1.73	-0.52	-1.42	-0.46	-3.84	-3.05
Pearson's linear correlation, r (p)				-0.36 (0.025)	-0.54 (0.00005)	-0.37 (0.01)	-0.52 (0.00025)	-0.30 (0.05)	-0.34 (0.025)
Spearman's rank correlation, R (p)				-0.43 (0.0025)	-0.53 (0.0001)	-0.42 (0.0025)	-0.50 (0.00025)	-0.35 (0.025)	-0.38 (0.01)
Kendall's rank correlation, τ (p)				-0.30 (0.0025)	-0.38 (0.0001)	-0.29 (0.005)	-0.36 (0.00025)	-0.25 (0.025)	-0.24 (0.025)
Goodman–Kruskal generalized correlation, γ (p)				-0.30 (0.0025)	-0.38 (0.0001)	-0.29 (0.005)	-0.36 (0.00025)	-0.25 (0.025)	-0.24 (0.025)

Notes. See Notes under Table S1.

© 2024 by the authors. Submitted for possible open access publication under the terms and conditions of the Creative Commons Attribution (CC BY) license (http://creativecommons.org/licenses/by/4.0/).
